# Tapping into the maize root microbiome to identify bacteria that promote growth under chilling conditions

**DOI:** 10.1186/s40168-020-00833-w

**Published:** 2020-04-18

**Authors:** Stien Beirinckx, Tom Viaene, Annelies Haegeman, Jane Debode, Fien Amery, Steven Vandenabeele, Hilde Nelissen, Dirk Inzé, Raul Tito, Jeroen Raes, Caroline De Tender, Sofie Goormachtig

**Affiliations:** 1 Department of Plant Biotechnology and Bioinformatics, Ghent University, 9052 Ghent, Belgium; 2 Center for Plant Systems Biology, VIB, 9052 Ghent, Belgium; 3 Plant Sciences Unit, Flanders Research Institute for Agriculture, Fisheries and Food (ILVO), 9820 Merelbeke, Belgium; 4 Aphea.bio NV, 9052 Ghent, Belgium; 5 Department of Microbiology and Immunology, Laboratory of Molecular Bacteriology, Rega Institute, KU Leuven, 3000 Leuven, Belgium; 6 Center for Microbiology, VIB, 3000 Leuven, Belgium; 7 Department of Applied Mathematics, Computer Sciences and Statistics, Ghent University, 9000 Ghent, Belgium

**Keywords:** Chilling temperatures, Root endosphere, Microbiome, Maize, Plant growth-promoting rhizobacteria, PGPR

## Abstract

**Background:**

When maize (*Zea mays* L.) is grown in the Northern hemisphere, its development is heavily arrested by chilling temperatures, especially at the juvenile phase. As some endophytes are beneficial for plants under stress conditions, we analyzed the impact of chilling temperatures on the root microbiome and examined whether microbiome-based analysis might help to identify bacterial strains that could promote growth under these temperatures.

**Results:**

We investigated how the maize root microbiome composition changed by means of 16S rRNA gene amplicon sequencing when maize was grown at chilling temperatures in comparison to ambient temperatures by repeatedly cultivating maize in field soil. We identified 12 abundant and enriched bacterial families that colonize maize roots, consisting of bacteria recruited from the soil, whereas seed-derived endophytes were lowly represented. Chilling temperatures modified the root microbiome composition only slightly, but significantly. An enrichment of several chilling-responsive families was detected, of which the *Comamonadaceae* and the *Pseudomonadaceae* were the most abundant in the root endosphere of maize grown under chilling conditions, whereas only three were strongly depleted, among which the *Streptomycetaceae*. Additionally, a collection of bacterial strains isolated from maize roots was established and a selection was screened for growth-promoting effects on juvenile maize grown under chilling temperatures. Two promising strains that promoted maize growth under chilling conditions were identified that belonged to the root endophytic bacterial families, from which the relative abundance remained unchanged by variations in the growth temperature.

**Conclusions:**

Our analyses indicate that chilling temperatures affect the bacterial community composition within the maize root endosphere. We further identified two bacterial strains that boost maize growth under chilling conditions. Their identity revealed that analyzing the chilling-responsive families did not help for their identification. As both strains belong to root endosphere enriched families, visualizing and comparing the bacterial diversity in these communities might still help to identify new PGPR strains. Additionally, a strain does not necessarely need to belong to a high abundant family in the root endosphere to provoke a growth-promoting effect in chilling conditions.

Video abstract.

## Background

Maize (*Zea mays L.*) is one of the most important staple crops worldwide with an annual production of approximately 1.4 billion tonnes in 2017 (www.fao.org). Due to its subtropical origin, cultivation of maize in the Northern hemisphere is rather challenging because of chilling spring temperatures [1]. These chilling temperatures, on average 17 °C during the day and 12 °C during the night, retard plant development, eventually resulting in yield losses [2–4]. As additional arable land is scarce, the proposed increase in maize needs to result from higher yields per area.

To boost plant growth, plant growth-promoting rhizobacteria (PGPR) have been proposed as an ecological additive under both normal and stress conditions [5–8]. As well in the plant roots, i.e. the root endosphere, and as in the closely surrounding soil, the rhizosphere, microbial communities are established that can affect plant fitness [6]. Deep-sequencing technologies have allowed us to obtain insight into the diversity of these microbial communities [9–13], demonstrating that the endo- and rhizospheres are occupied by a vast selection of microbes that are recruited from the soil. The plant compartment plays an important role in this process: bacterial community compositions gradually change from the soil toward the inside of the root [10–12]. The root endosphere-colonizing microbes are expected to engage in robust interactions with the plant roots, but they could also have been acquired by vertical transmission of seed microbes besides recruitment from the soil [14–16].

Varying environmental cues, including pathogen attack [17, 18] and abiotic stresses, such as drought and salt [19, 20], modify the microbial composition of the root endosphere and rhizosphere. As a result, plants have been proposed to select a microbiome that provides the best plant fitness [17–19].

Even though chilling temperature is one of the major abiotic stresses many plants have to cope with during their life cycle, especially when grown in temperate regions, its effect on the microbiome has not been investigated yet, either in maize or other plants. More effort has been put in the identification of particular PGPR strains that can alleviate chilling stress in plants. Several psychrotolerant bacterial strains belonging to different families, such as *Burkholderiaceae*, *Bacillaceae*, and *Pseudomonadaceae,* have been identified that can help plants such as *Arabidopsis thaliana*, rice (*Oryza sativa)*, wheat (*Triticum* sp.), tomato (*Solanum lycopersicum*), and grapevine (*Vitis vinifera*), to cope with chilling stress [21–26]. However, to our knowledge, bacteria that can promote maize growth under chilling stress have not been identified yet.

As chilling stress restricts maize growth, research on its impact on the microbiome might guide the selection of possible growth-promoting bacteria. To test this hypothesis, in independent repeats, we identified the main endosphere microbiome of maize roots and analyzed the effects of chilling temperatures on its composition. In parallel, a bacterial collection of maize endophytes was established and screened for growth promotion of juvenile maize under chilling stress.

## Materials and methods

### Microbiome experimental design

Five experiments (experiments I to V) were set up with different objectives (Table 1). For all experiments, surface-sterilized hybrid uncoated maize seeds (LG30.217, Limagrain, Saint Beauzire, France) were used. Briefly, seeds were washed 5 min with sterile water, 2 min with 70% (v/v) ethanol, 20 min with a bleach solution (29 ml sterile water, 15 ml NaClO, 12–13% (v/v) stock solution, and 1 ml Tween 20), five times for 15 min with sterile water, then air-dried, and stored at 4 °C until further use.

**Table 1:** Experimental setups

Experiment	Growth conditions	Compartment studied	Main objective
I	Maize in the field	Bulk soil vs. root endosphere	Define main root microbiome (field setup)
II	Maize in field-soil filled pots	Bulk soil vs. root endosphere	Define main root microbiome (growth chamber setup)
III	Maize in field-soil filled pots and in vitro	Root endosphere of plants grown in soil- vs. in vitro-	Define origin of root microbiome = seed or soil
IV	Maize in field-soil filled pots under control and chilling conditions	Root endosphere of maize grown under control vs. chilling conditions	Define chilling-responsive families
V	Maize in field-soil filled pots under control and chilling conditions	Root endosphere of maize grown under control vs. chilling conditions	Define chilling-responsive families

In experiment I, seeds were sown according to common agricultural practices in an experimental field with sandy loam soil (United State Department of Agriculture classification) (50° 58′ 41′′ N, 3° 46′ 47.28′′ E; Merelbeke, Belgium). Approximately after 3 weeks of growth, until development of the sixth leaf, bulk soil samples and root endosphere samples of five plants were collected as described below.

In experiment II, top soil was collected from the same field as that for experiment I and sieved before use. Soil characteristics were determined at the beginning of the experiment (Additional file 1: Table S1). Twelve pots were filled with freshly collected soil, six pots were kept uncultivated and used for bulk soil sampling, whereas in six other pots, sterilized maize seeds were sown and the root endosphere was sampled after development of leaf 6 (4 weeks of growth). All pots were maintained in the growth room under controlled conditions (16 h/8 h light/dark regime, 21 °C) and kept well-watered throughout the time of the experiment.

In experiment III, as in experiment II, top soil from the experimental field was collected and 12 pots were filled with the collected soil. Again, six were kept bare for bulk soil samples and in the six remaining pots, maize was sown in soil to identify the root endosphere of soil-grown plants after 2 weeks of growth. Additionally, six sterilized seeds were sown in transparent plastic boxes filled with Hoagland’s solution (0.945 g/l Ca(NO_3_)_2_.4H_2_O, 0.506 g/l KNO_3_, 0.136 g/l KH_2_PO_4_, 0.493 g/l MgSO_4_.7H_2_O, 2.5 ml/l Fe-EDTA stock (5.56 g FeSO_4_.7H_2_O, 7.46 g/l Na-EDTA)) supplemented with plant agar. Due to space limitation in the boxes, samples were collected after 2 weeks of growth under constant conditions (16 h/8 h light/dark regime, 21 °C).

In experiments IV and V, ten maize plants were grown as in experiment II for 5 weeks under chilling (16 h/8 h light/dark regime at 17 °C/12 °C) or control temperature conditions (16 h/8 h light/dark regime, 21 °C) and kept equally well-watered throughout the time of the experiment. The sampling of the field soil for experiment IV was repeated in experiment V, but after a 1-year gap.

To ensure that bulk soil and root endosphere samples were treated similarly, both were washed before DNA extraction. For the bulk soil, samples were washed in phosphate-buffered saline (PBS) solution for 20 min and centrifuged at 3220×*g* for 20 min to ensure that all bacteria and soil particles were dissolved in the pellet; however, mostly soil-particle-sorbed microbes will be captured. The supernatant was removed and the remaining soil pellet was frozen in liquid nitrogen and stored at − 80 °C. For the root endosphere sample collection, adhering soil was removed by shaking the roots vigorously. Roots were washed twice in PBS by shaking in 500-ml sterile flasks with 50 ml PBS for 20 min, sonicated (10 min of 30-s cycles at 4000 Hz) to remove remaining sticking microorganisms, flash-frozen in liquid nitrogen, and stored at − 80 °C. Roots were ground in liquid nitrogen before DNA extraction. DNA was isolated from all collected samples with the DNeasy PowerSoil DNA kit (QIAGEN, Hilden, Germany), whereafter the V4 region (515F-806R) of the 16S rRNA gene was amplified as proposed by The Earth Microbiome Project (www.earthmicrobiome.org). Reverse bar-coded primers were used to amplify the V4 region in triplicate with the iProof High-Fidelity PCR Mix (Bio-Rad, Hercules, CA, USA) with 30 cycles of amplification at 55 °C for 30 s. Pooled PCR products of the triplicate samples were purified with Agencourt AMPure XP magnetic beads (Beckman Coulter, Pasadena, CA, USA) and DNA concentration was measured with a Qubit fluorometer (Thermo Fisher Scientific, Waltham, MA, USA). Based on previous experiments, a high amount of 16S rRNA gene reads from chloroplastic and mitochondrial origin was expected in the root endosphere, thus the bulk soil and root endosphere samples were unequally pooled in a 1:5 ratio. Sequencing was done on an Illumina MiSeq platform (v3, 2x300 bp) according to the Illumina protocols (VIB, Nucleomics Core, Leuven, Belgium). The raw sequence reads were deposited and are available for download at the NCBI sequence reads archive (SRA) with project number PRJNA523518 (experiments I, II, and III) and PRJNA524079 (experiments IV and V).

The sequencing reads were demultiplexed and primers were removed by the sequencing provider. The sequence read quality was checked by FastQC [27]. Read quality and length trimming were done with DADA2, using the default quality score (truncQ = 2); forward reads were truncated at 240 bp and reverse reads at 200 bp (truncLen = c(240,200)) and a maximum of three expected errors both in the forward and in the reverse reads was allowed (maxEE = c(3,3)). Determination of amplicon sequence variants (ASVs) and taxonomy assignments were done by means of the standard DADA2 pipeline v1.6.0 [28]. After ASV determination, each sample had approximately 100,000 quality checked read (Additional file 1: Table S3). The resulting count table was filtered to remove low abundant ASVs (less than two counts/ASV in at least five samples), reducing the read count per sample to an average of 40,000 reads (Additional file 1: Table S3). Taxonomy was assigned according to the SILVA Database version 128 [29]. Based on the taxonomy assignment, reads belonging to chloroplasts (Class *Chloroplast*) and mitochondria (Order *Rickettsiales*) were removed, reducing the number of reads by an average of 5% and 80% in the bulk soil and root endosphere, respectively. The resulting ASV count tables were used for further statistical analysis.

### Bacterial collection and screening assay

To obtain a collection of endophytic bacterial strains, the maize plants were grown in soil collected from the experimental field for 4–6 weeks under chilling temperature conditions (16 h/8 h light/dark at 17 °C/12 °C). To ensure isolation of endophytes, maize roots were washed and sterilized as follows: three times for 5 min with sterile water, 30 s with 70% (v/v) ethanol, 3 min with bleach solution (12% [v/v] sodium hypochlorite; Chem-Lab, Zedelgem, Belgium), and five times with sterile water for 5 min. Roots were crushed on ice with sterile mortar and pestle in PBS, pushed through a 70-μm cell strainer, and diluted 1/100 and 1/1000 with PBS.

Diluted suspensions were plated on three different commonly used bacterial media TSB, King’s B, and R2A (Additional file 1: Table S2) and incubated for maximum 2 weeks. Growing colonies were selected, streaked until pure cultures, and identified based on 16S rRNA gene using universal 16S rRNA primers: 27F (AGAGTTTGATCMTGGCTCAG) and 1492R (GGTTACCTTGTTACGACTT). DNA was extracted with alkalytic lysis [30]. In total, 1 μl of supernatant DNA was used for PCR amplification with 0.2 μl polymerase (IProof^TM^ High Fidelity DNA Polymerase; Bio-Rad), 1 μl of 10 μM forward primer, and 1 μl of 10 μM reverse primer, 0.4 μl 10 mM dNTP mix, and 4 μl buffer 5× IProof^TM^ buffer (Bio-Rad). PCR conditions were denaturation at 98 °C for 3 min, 30 cycles of 98 °C for 10 s, 55 °C for 20 s, and 72 °C for 45 s, and a final elongation at 72 °C for 5 min. The resulting PCR products were purified with the GeneJET PCR Purification Kit (Thermo Fischer Scientific) and sequenced by Sanger sequencing (Eurofins Genomics, Ebersberg, Germany). Forward and reverse sequences were merged by means of the CLC Main Workbench 7 (CLC Bio-QIAGEN) and taxonomy was assigned to the resulting sequences with the SILVA database version 128.

Of the bacterial collection, isolates representing either the main root endosphere microbiome families identified in experiments I and II, the chilling-responsive endosphere families identified in experiments IV and V, or other endophytic families were selected. In total, 28 of the 282 bacterial isolates in the collection were tested for maize growth promotion. Maize seeds were surface-sterilized as described above and pre-germinated for 48 h in the dark at 24 °C on 1% (w/w) agar plates. The effect of each strain was tested in two or three repeats. For each repeat, 15 seedlings were inoculated by shaking in a bacterial solution for 3 h. The different bacterial isolates were grown in liquid medium and diluted to OD_600_ 0.02 with PBS buffer. For each experiment, a mock-inoculated treatment was included, for which 15 seedlings were inoculated with PBS buffer for 3 h before sowing. Bacterial- and mock-inoculated seedlings were sown in sand/perlite (50/50 volume %) and cultivated under controlled growth conditions in the growth chamber (16 h/8 h light/dark regime at 17 °C/12 °C). The 15 plants were cultivated in separate pots (square pots: 7 × 7 × 8 cm); per treatment, all pots were put together on one tray. The entire tray was watered (500 ml) every 2 days, while nutrients (150 ml Hoagland’s solution for 15 plants) were added once a week. After 30 days of growth, plants were harvested and the fresh total (root and shoot) weight was analyzed. Additionally, the effect of the two identified PGPR strains, RHG5 and RHG12, was analyzed under control temperature conditions. The experimental setup was the same as for the screening under chilling conditions, with the exception that the growth conditions differed, namely 16 h/8 h light/dark regime and 21 °C, and the plants were harvested after 25 days of growth.

### Statistical analysis

All statistical analyses were done in R (version 3.4.0). For the microbiome experiments, the ASV count tables, generated by DADA2 [28], were filtered to remove low abundant ASVs. ASVs with a count number of 2 in at least five samples were retained. The multivariate analysis was done using the R package vegan (version 2.0-10) [31]. The dissimilarity matrix, based on the Bray-Curtis dissimilarity index, was calculated from the ASV tables as generated by DADA2 [28]. By means of the betadisper function, the homogeneity of the variances was checked on this dissimilarity matrix. Further, the significance of sample type (e.g., bulk soil, root endosphere of soil-grown and in vitro-grown plants) or chilling treatment and experiment were analyzed with PERMANOVA, in which the Bray-Curtis dissimilarity index matrix was used as input. The temperature experiments IV and V were considered as biological repeats. To test the effect of the experiment, the combined ASV table was analyzed with PERMANOVA, showing ‘biological repeat’ as a significant factor (*P* < 0.001) that shapes the bacterial communities. Hence, the statistical analyses were done separately for each experiment. Additionally, PERMANOVA analysis demonstrated a significant interaction effect in both experiments between sample type (bulk soil and root endosphere) and temperature (control and chilling) (*P <* 0.001).

The differential abundance was assessed with the likelihood-ratio tests. Analyses were done at the levels of ASV, family, and phylum, using the ASV counts and taxonomy for the latter two. The data were normalized based on the library size of the count table, resulting in analysis of the relative abundances. All these analyses were done with the edgeR package, version 3.18.1 [32].

In the screening assay, the statistical differences in growth promotion due to bacterial inoculation were analyzed by general linear models, after the assumptions of linearity, normality, and homogeneity of the data had been checked. The experiment was repeated two or three times and an interaction effect between treatment and repeat was identified (*P* < 0.05). Therefore, the growth-promoting effects of bacterial strains were examined for each repeat separately. The fresh total (root and shoot) weight total was compared between bacteria- and mock-inoculated plants with a two-sided Student’s *t* test (analysis done in R).

## Results

### Identification of the main root microbiome families of maize grown in field soil

**Fig. 1.**
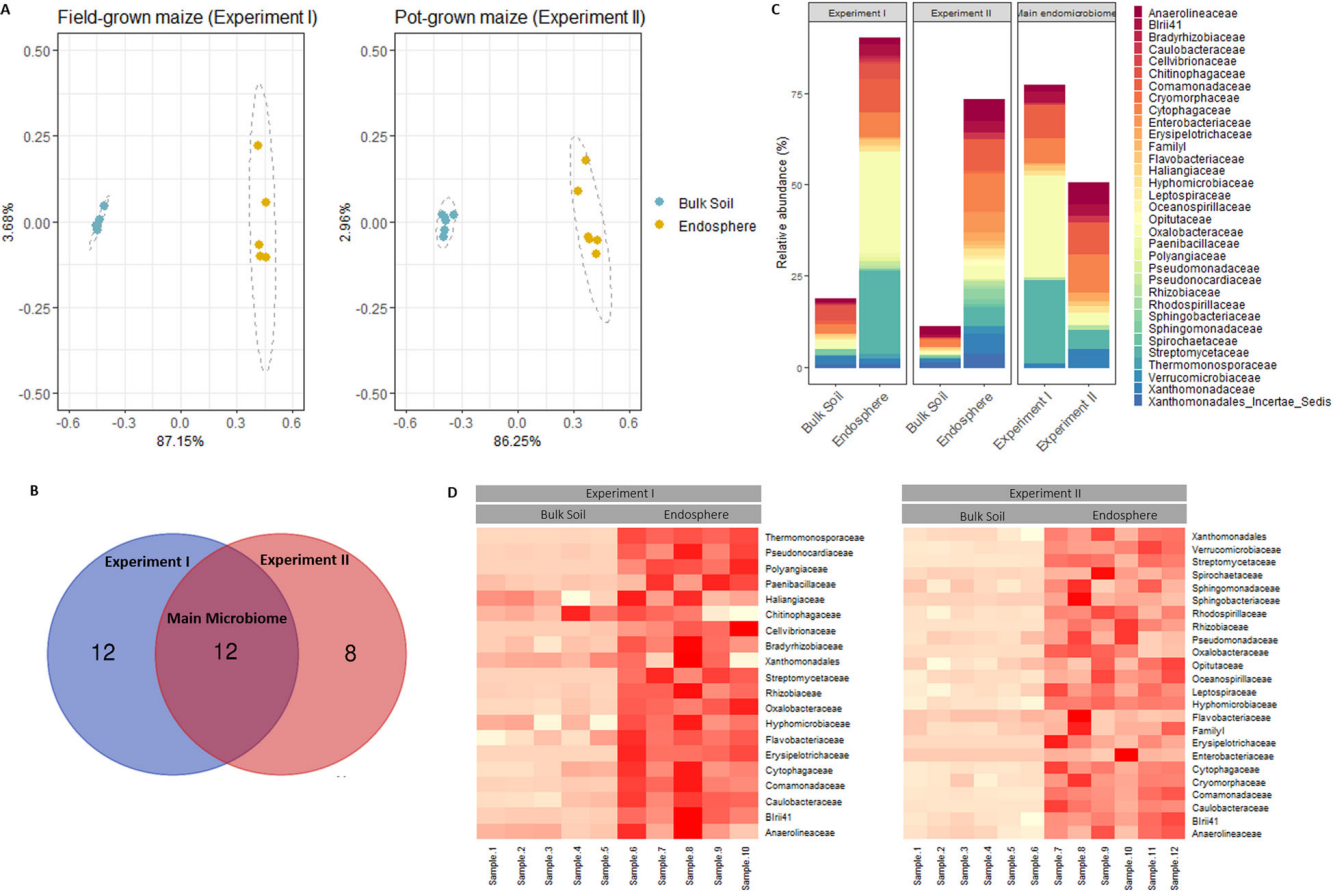
Identification of the main maize root endosphere families of field-grown and pot-grown maize. **a** Principal coordinate analysis of the microbial communities in bulk soil and endosphere in field- and pot-grown maize (experiments I and II). PCoA plots are based on Bray-Curtis dissimilarity indices. **b** Analysis of the bacterial communities in both experiments at the family level. The first two panels show the mean relative abundance of families (> 0.5% in the root endosphere) in bulk soil and endosphere. The third panel shows the relative abundance of the identified main microbiome families (highly abundant and enriched in both experiments) in the root endosphere. **c** Overlap of the enriched (*P* < 0.05) and abundant families (relative abundance > 0.5%) in the root endosphere of experiments I (blue) and II (red) representing the families of the main microbiome. **d** Heatmap of the enriched (*P* < 0.05) and abundant families (relative abundance > 0.5%) in the root endosphere of each experiment. Bulk soil and root endosphere samples are presented separately

To identify the main bacterial community of the maize root endosphere (hereafter designated root endosphere), grown in field soil, two experiments were carried out. In experiments I and II (Table 1), maize was grown in the field and in field soil-filled pots, respectively. Sequences assigned to chloroplasts and mitochondria were removed from quality-filtered reads, resulting in ASV count tables containing 1013 unique ASVs in experiment I and 820 in experiment II (Additional file 1: Table S3). Sample type, i.e., bulk soil or root endosphere, was the major variance driver (87.15% and 86.25% for experiments I and II, respectively) for the differences in the bacterial community composition as found by the PERMANOVA analysis (*P* < 0.001) and illustrated in the Bray-Curtis dissimilarity-based principal coordinate analysis (PCoA) plot (Fig. 1a).

In both maize grown in the field (experiment I) and in pots (experiment II), five highly abundant phyla were significantly enriched (*P* < 0.001) in the root endosphere compared to the bulk soil: *Proteobacteria*, *Bacteroidetes*, *Chloroflexi*, *Firmicutes*, and *Actinobacteria* (Additional file 1: Table S4)*.* Two dominating phyla in the pot-grown root endosphere were *Spirochaetae* and *Cyanobacteria*, phyla that were either not detected or at a very low abundance in the root endosphere of field-grown plants (Additional file 1: Table S4).

To characterize the main root endosphere microbiome, we compared the results of both field- and pot-grown plants. The main microbiome was defined as the bacterial families that were noteworthy abundant (relative abundance > 0.5%) and significantly enriched (*P* < 0.05) in the root endosphere of both experiments (Fig. 1b, d). We detected 12 families that met these requirements, namely *Anaerolineaceae*, *Blrii41*, *Caulobacteraceae*, *Comamonadaceae*, *Cytophagaceae*, *Erysipelotrichaceae*, *Flavobacteriaceae*, *Hyphomicrobiaceae*, *Oxalobacteraceae*, *Rhizobiaceae*, *Streptomycetacea*e, and an unclassified *Xanthomonodales* family (Fig. 1c, d; Additional file 1: Table S4). Although the same enrichments were found in both experiments, the abundance of the bacterial families differed for the two root endosphere communities. The identified main microbiome families accounted for 76.94% and 45.97% of the bacterial community of the root endosphere of field-grown (experiment I) and pot-grown (experiment II) plants (Fig. 1c), respectively. The most striking discrepancy occurred in the abundance of *Actinobacteria*. In the root endosphere of field-grown maize (relative abundance ± SE; 27.18 ± 2.91%), the relative abundance of this phylum was 5-fold higher than in the pot-grown maize root endosphere (5.67 ± 0.64%) (Additional file 1: Fig. S1). In both experiments, almost all actinobacterial ASVs were assigned to one family, the *Streptomycetaceae* (Additional file 1: Fig. S1). The enrichment was strong under both conditions (logFC 9.4 and 7.3 for experiments I and II, respectively) and the initial abundance in the bulk soil was low (0.035 ± 0.02% and 0.05 ± 0.01% for experiments I and II, respectively). Thus, it is tempting to speculate that the growing conditions, i.e., field or pot, affect the *Streptomycetaceae* abundance.

Although the abundance of the *Proteobacteria* did almost not vary between the pot-grown (46.09 ± 3.52%) and field-grown (52.42 ± 2.14%) root endosphere, the abundance of the proteobacterial family *Oxalobacteraceae* differed, accounting for almost 30% (27.77 ± 3.16%) of the root endosphere of field-grown maize and less than 4% (3.62 ± 0.35%) of the root endosphere of pot-grown maize. In comparison, the abundance of this family in the field bulk soil (2.6 ± 0.76%) was higher than in the pot bulk soil (0.14 ± 0.01%), whereas the fold changes between the experiments were comparable (logFC 4.26 vs. 4.86) (Additional file 1: Table S4).

To conclude, the growth conditions in field soil and field soil-filled pots affected the root endosphere microbiome. Despite differences in abundances, the recurring enrichment pattern remained similar and resulted in the identification of a main root microbiome of maize grown in Belgian field soil of 12 different families.

### Contribution of the seed-inherited root microbiome to the root microbiome

The root endosphere is colonized by soil-inhabiting bacteria, i.e., bacteria that were recruited through horizontal transmission from the soil, and by seed-inhabiting bacteria that colonize the inner tissues of the seed and might be potentially considered vertically transmitted [14, 15, 33, 34]. In view of identifying bacterial strains for application via seed coating, e.g., the inoculation of bacteria at the outside of the seed, it is important to make out whether bacteria can colonize the root endosphere from the surrounding soil or via the seeds. To this end, we set up a third experiment (experiment III; Table 1). Here, we compared bulk soil samples, root endosphere samples of 2-week-old maize seedlings grown in vitro under gnotobiotic soil-free conditions, and root endosphere samples of seedlings grown in field soil-filled pots.

**Fig. 2:**
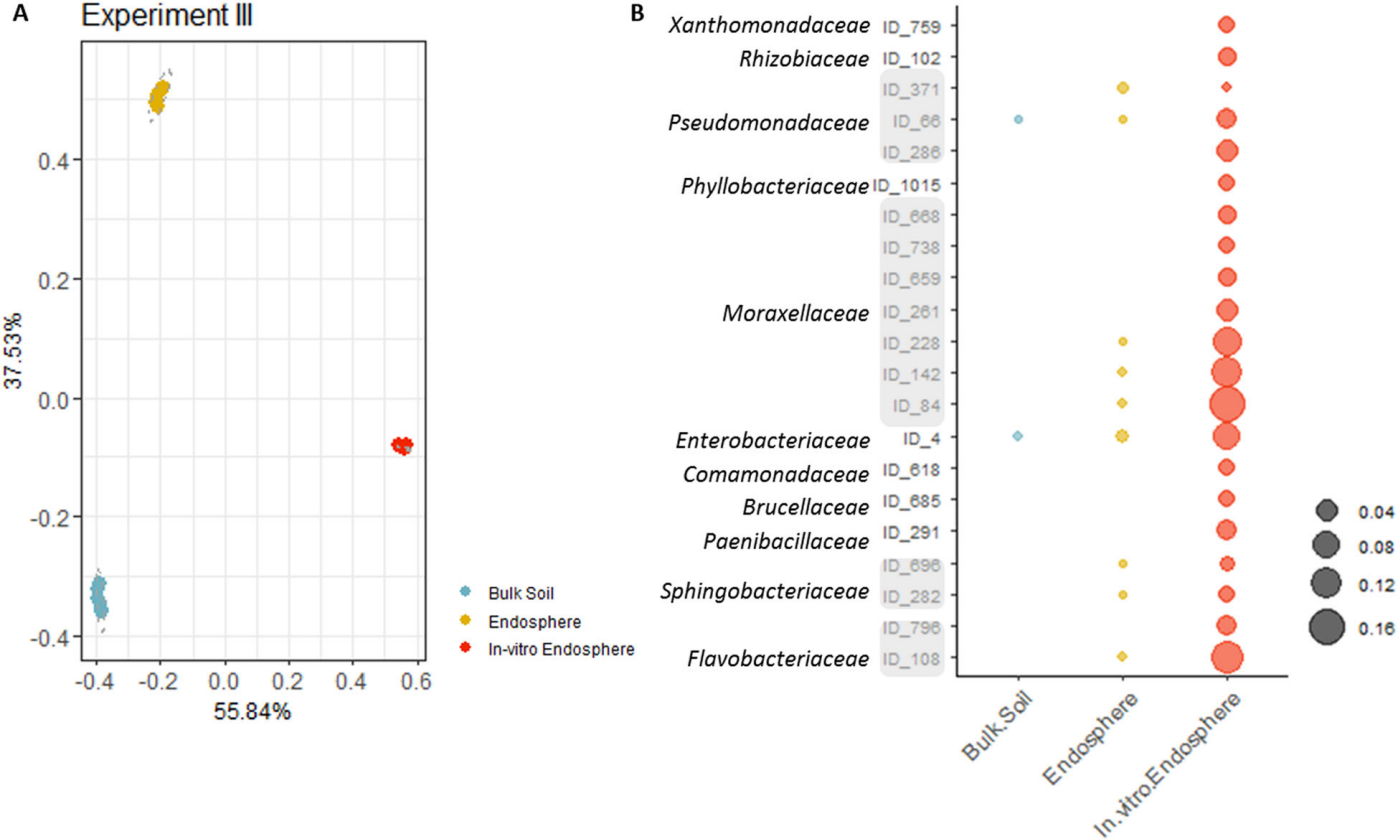
Contribution of the seed-inherited root microbiome to the maize root microbiome. **a** PCoA plot based on Bray-Curtis dissimilarity indices of the microbial communities in bulk soil and root endosphere of soil-grown and of in vitro-grown maize in experiment III. **b** Presence and abundance of the 21 ASVs belonging to 11 different families, detected in the root endosphere of in vitro-grown maize plants and compared with the abundance of the root endosphere of soil-grown maize and bulk soil samples

PERMANOVA analysis showed that sample type explained most of the variation in the bacterial community structure as illustrated in the PCoA plot (*P* < 0.01; based on Bray-Curtis distances) (Fig. 2a). Besides distinct clustering of the bulk soil samples as in experiments I and II, the root endosphere of the plants grown in soil and in vitro also clustered separately. This clustering can be partially explained by the different nature of the growth substrate of the plants, i.e., field soil vs. Hoagland’s solution provided with agar. Besides the substrate, the clustering can also be affected by the strong difference in read counts after filtering out the reads assigned to chloroplasts and mitochondria of the root endosphere of soil-grown and in vitro-grown maize; in total, 10% and 1%, respectively, of the reads were retained (Additional file 1: Table S3). As a result, the bacterial diversity in the root endosphere of the in vitro-grown plants was very low (Additional file 1: Fig. S2).

In total, 21 bacterial ASVs were detected in the root endosphere of in vitro-grown plants, belonging to three different phyla: *Bacteroidetes* (four ASVs; relative abundance ± SE, 19 ± 1.23%), *Firmicutes* (one ASV; 3 ± 0.34%), and *Proteobacteria* (16 ASVs; 76 ± 1.24%), represented by 12 different families (Fig. 2b; Additional file 1: Table S5). Nine of these ASVs were among the 426 ASVs detected in the bulk soil bacterial community retained after filtering out chloroplast, mitochondrial, and low abundant reads. The family *Moraxellaceae* (*Proteobacteria*) contained seven and three ASVs in the root endosphere of in vitro or in soil-grown plants, respectively. However, the family was not detected in the bulk soil indicating that these ASVs most probably colonized the root endosphere through the seeds (Fig. 2b; Additional file 1: Table S5). The second most abundant family in the root endosphere of in vitro-grown plants was the *Flavobacteriaceae* (two ASVs; 16.2 ± 0.63%), with one ASV representing 13% that also occurred in the root endosphere of soil-grown plants. Several *Flavobacteriaceae* were detected in the bulk soil as well, but were not the same ASVs as those found in the root endosphere of in vitro-grown plants. Two of the ASVs detected in the root endosphere of in vitro-grown plants were present both in the bulk soil and root endosphere of soil-grown plants, one belonging to the *Pseudomonadaceae* family and one belonging to the *Enterobacteraceae* family (Fig. 2b). Whether these bacteria came through the seeds or via the soil into the root endosphere cannot be distinguished. Based on these data, we can assume that the root endosphere was acquired mainly from the surrounding soil microbial community, although transmission through the seeds occurred as well.

### The effect of chilling temperature on the maize root endosphere microbiome

**Fig. 3:**
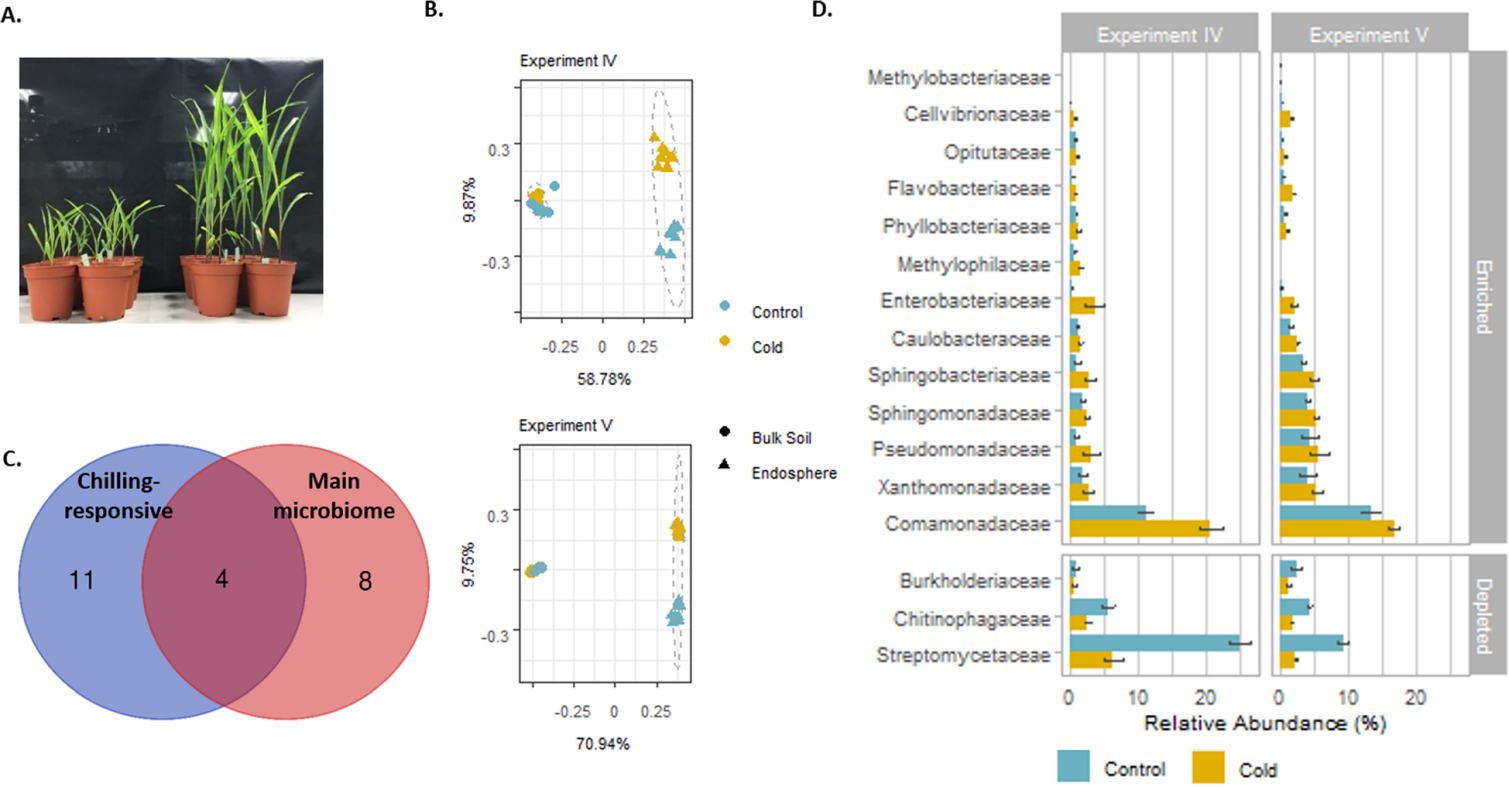
Bacterial community shifts upon chilling temperature treatment in experiments IV and V. **a** Four-week-old maize plants grown in field soil. Plants on the left and the right are grown under chilling (16 h/8 h light/dark regime and 17 °C/12 °C) and under normal (16 h/8 h light/dark regime and constant 21 °C) temperature conditions, respectively. **b** Principal coordinate analysis of the microbial communities in bulk soil and root endosphere. Effects of the variables temperature and compartment are given by differences in color and shape, respectively. PCoA plots are based on Bray-Curtis dissimilarity indices. **c** Overlap between the chilling-responsive families in experiments IV and V and the main microbiome. **d** Root endosphere bacterial families enriched (top) and depleted (bottom) in chilling experiments IV and V (relative abundance > 0.5%). The relative abundance of the families is presented; error bars represent the standard error

To analyze the effect of chilling temperature on the root endosphere, we set up experiments IV and V (Table 1; Fig. 3a). In both experiments, maize was grown in field soil-filled pots under either ambient or chilling temperature conditions. After filtering and removal of plant-related reads, 1137 and 962 unique ASVs were detected for experiments IV and V, respectively (Additional file 1: Table S3). Besides the expected shift in community due to sample type (see experiments I and II), we observed a significant interaction effect between temperature and sample type, indicating that the bacterial taxonomical changes upon chilling varied between the bulk soil and the root endosphere (*P <* 0.001). Both in the bulk soil and in the root endosphere, the temperature had a significant effect on the bacterial communities (Additional file 1: Table S6). As seen in the PCoA plots based on Bray-Curtis distances (Fig. 3b), the shift in the microbial community of the chilling-treated bulk soil was smaller in both experiments than that in the root endosphere bacterial communities (Fig. 3b; Additional file 1: Fig. S3 and Table S6). In the root endosphere, temperature was responsible for more than 40% of the detected variance (41.43% and 48.77% in experiments IV and V, respectively), whereas in the bulk soil samples, the variation differed less (38.01% and 34.48% in experiments IV and V, respectively) (Additional file 1: Fig. S3 and Table S6). In the bulk soil of experiment IV, chilling stress had the highest influence on the *Chitinophagaceae*, increasing with a logFC of 1.6, from 1.6 ± 0.19 to 4.6 ± 0.52% under chilling conditions (*P* < 0.001). In the bulk soil of experiment V, chilling temperatures resulted in a major increase in the abundance of *Blastocatellaceae*, from an average of 14.08 ± 0.63% to 28.20 ± 0.91% (Additional file 1: Table S7). Thus, no recurring pattern in community shifts at the family or phylum level was demonstrated in the bulk soil.

In contrast, the root endosphere differed significantly between the control and chilling - grown maize plants. At phylum level, a significant decrease in *Actinobacteria* in the root endosphere upon chilling was detected in both experiments IV and V (*P <* 0.001) (Additional file 1: Table S7). Additionally, we identified 15 families (relative abundance > 0.5%) that consistently responded in the root endosphere under chilling conditions, of which 12 were enriched and three were depleted in both experiments. We compared the so-called chilling-responsive families with the identified main microbiome and detected an overlap of four bacterial families (Fig. 3c). The 12 enriched chilling-responsive families (relative abundance > 0.5%) were *Enterobacteriaceae*, *Pseudomonadaceae*, *Sphingobacteriaceae*, *Sphingomonadaceae*, *Methylophilaceae*, *Phyllobacteriaceae*, *Opitutaceae*, *Cellvibrionaceae*, *Flavobacteriaceae*, *Comamonadaceae*, *Caulobacteraceae*, and *Xanthomonadaceae* (Fig. 3d; Additional file 1: Table S7). Furthermore, three other chilling-responsive families were recurrently depleted in the chilling versus the control root endosphere, i.e., *Chitinophagaceae, Burkholderiaceae*, and *Streptomycetaceae* (Fig. 3d Additional file 1: Table S7)*.* The latter is part of the *Actinobacteri*a phylum that also significantly decreased in the root endosphere upon chilling and consisted primarily of *Streptomycetaceae*. This family decreased from an average abundance of 25 ± 1.52% in the control to 6 ± 1.37% in the chilling root endosphere in experiment IV and from 9.3 ± 0.77 to 2.3 ± 0.22% in experiment V (Fig. 3d; Additional file 1: Table S7).

In conclusion, two independent experiments in which the same field soil was used showed that chilling temperatures affected the microbial communities in the root endosphere in a recurring pattern, both causing enrichment and depletion of certain chilling-responsive families and, to a lesser extent, in the bulk soil environment.

### Screening for growth-promoting bacterial strains of maize under chilling temperatures

**Fig. 4:**
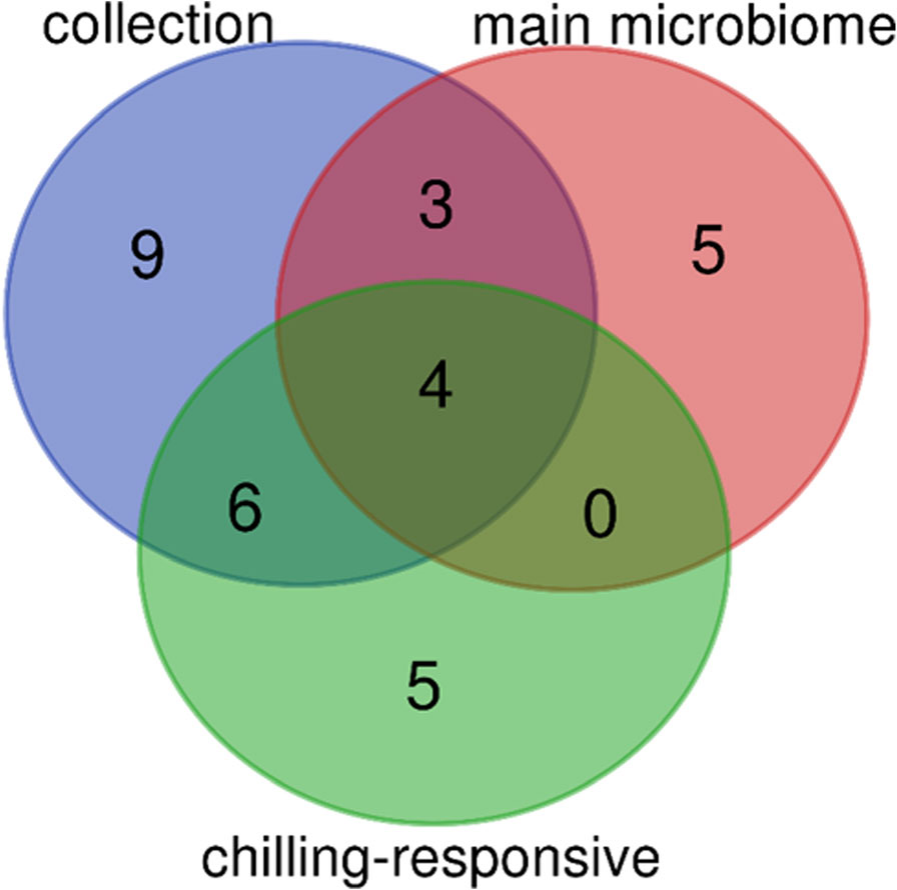
Bacterial collection of maize endophytes. The number of detected families in the collection, the families in the main microbiome, and chilling-responsive families is given together with the overlap of the three groups

We hypothesized that under chilling temperatures, the root endosphere might recruit bacterial taxa that could trigger adaptation to this stress and promote plant growth. To this end, a bacterial collection of 282 maize root endophytic bacterial strains was established from plants grown in chilling temperatures (Additional file 2: Table S8A, B, C). The collection represented four well-known root endosphere phyla, i.e., *Proteobacteria* (*n* = 178), *Actinobacteria* (*n* = 29), *Bacteroidetes* (*n* = 14), and *Firmicutes* (*n* = 61) as well as 22 different endophytic families (Additional file 2: Table S8A). The bacterial collection represents more than half (*n* = 7) of the families of the identified main microbiome, i.e., *Flavobacteraceae*, *Comamonadaceae*, *Caulobacteraceae*, *Streptomycetaceae*, *Rhizobiaceae*, *Oxalobacteraceae*, and *Cytophagaceae* (Fig. 4)*.* Four families that are both identified in the main microbiome and chilling-responsive are present in the collection: *Flavobacteraceae*, *Comamonadaceae*, *Caulobacteraceae*, and *Streptomycetaceae* (Figs. 3c and 4)*.* Additionally, six other chilling-responsive families could be isolated, i.e., *Enterobacteraceae*, *Pseudomonadaceae*, *Burkholderiaceae*, *Xanthomonadaceae*, *Sphingomonadaceae*, and the *Sphingobacteriaceae* (Fig. 4)*.*

**Fig. 5:**
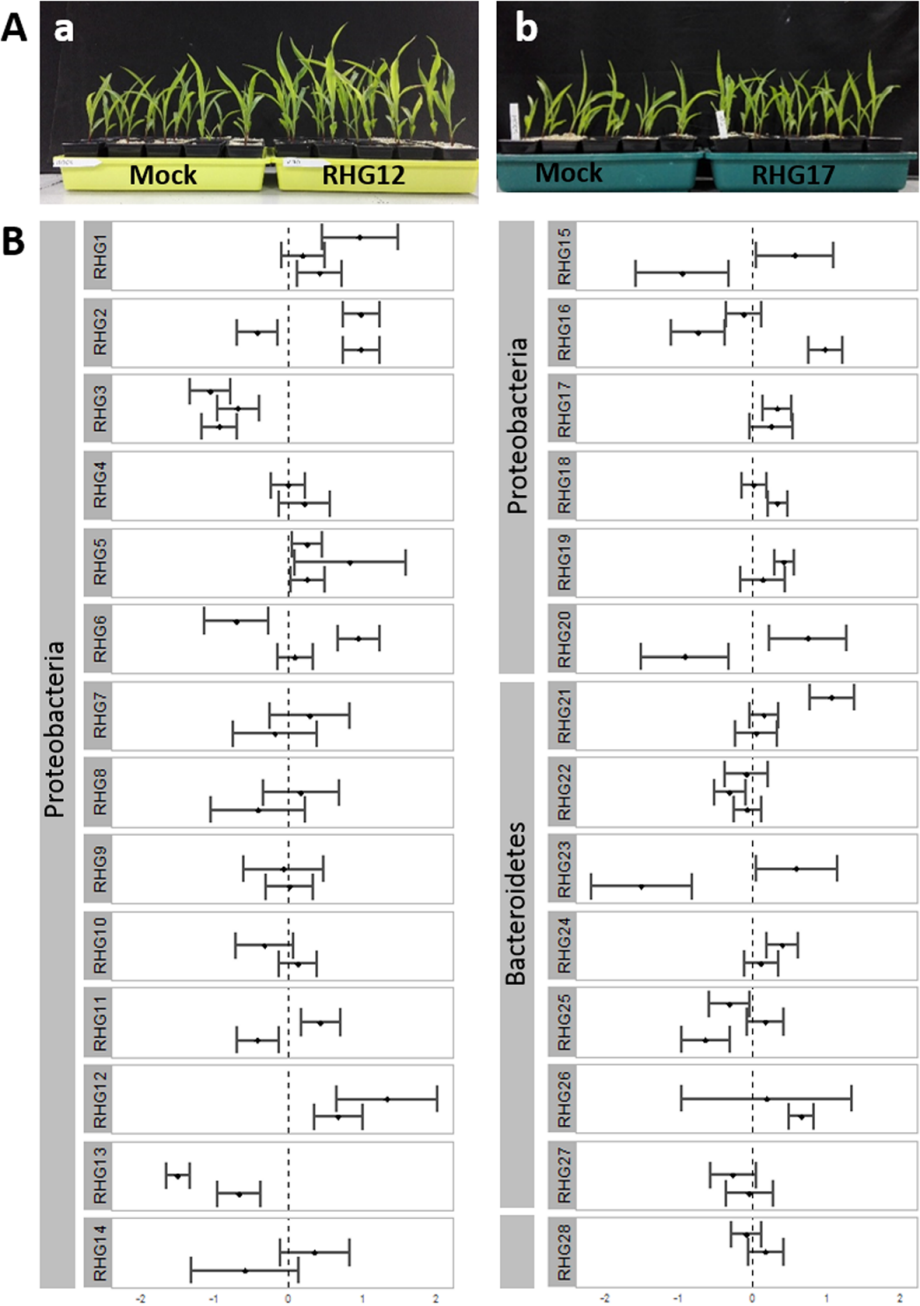
Screening assay for the effect of different bacterial isolates. **a** Inoculated (right tray) and mock-inoculated (left tray) juvenile maize plants grown for 30 days under chilling conditons (16 h/8 h light/dark regime and 17 °C/12 °C) (**a**) treated with RHG12 (*Pseudoduganella* sp.) (right tray) and (**b**) treated with RHG17 (*Rhizobium* sp.) (right tray). **b** Different bacterial isolates screened for growth-promoting effects on juvenile maize grown under chilling stress conditions. In total, 28 different isolates belonging to three different phyla were screened. Total fresh weights of bacterial- and mock-inoculated (*n* = 15) plants were measured and compared in two or three repeats. The figure illustrates the 95% confidence interval, based on a two-sample Student’s *t* test, of the treated plants compared with the mock-inoculated control. When the confidence interval does not cross the dashed line at zero, the effect is significant

A subset of the bacterial collection was screened for their growth-promoting effects on the total fresh weight of juvenile maize grown under chilling conditions (Fig. 5; Additional file 1: Table S9 and Fig. S4). The subset used for screening contained 14 strains belonging to main microbiome families, 11 strains belonging to the chilling-responsive families, and three strains of families enriched in the root endosphere but low abundant. Growth promotion of the bacterial strains was evaluated based on the fresh total weight (root and shoot), of bacteria-inoculated versus mock-inoculated maize. Two strains showed consistent growth-promoting effects over the different repeats: RHG5 (*Bradyrhizobiaceae*–*Bosea* sp.) and RHG12 (*Oxalobacteraceae*–*Pseudoduganella* sp.) with an average fresh plant weight increase of 18% and 33%, respectively (Fig. 5; Additional file 1: Table S9 and Fig. S4). The *Oxalobacteraceae* were identified as a main microbiome family, whereas the *Bradyrhizobiaceae* were enriched in the root endosphere, albeit at a low relative abundance, but neither were identified as chilling-responsive families.

Additionally, to investigate whether the two identified PGPR strains specifically promoted maize growth under chilling temperatures or whether they were robust growth strains, we tested their effect under control temperature conditions. Neither RHG5 nor RHG12 consistently affected maize growth under these control temperatures (Additional file 1: Fig. S5).

Furthermore, to detect whether the PGPR selection would be improved at the sequence level, we compared the identified PGPR strains of experiments IV and V at sequence level. RHG12 had a 100% similarity with ASV ID_10, that was highly abundant in the chilling and control root endosphere of both experiments IV and V and was strongly enriched compared to the bulk soil (Additional file 1: Table S10). In contrast, for RHG5, we could not detect an ASV with a 100% similarity in the filtered count table.

Thus, selection of bacterial strains belonging to chilling-responsive families is not enough to detect PGPR; nevertheless, families enriched in the root endosphere might be a good criterium to pick strains for large screening assays.

## Discussion

Chilling temperatures are one of the major abiotic stress factors affecting maize growth in the Northern hemisphere, with yield decrease as a consequence [3]. The use of growth-promoting bacteria for maize could boost maize growth under chilling conditions, but are, to our knowledge, not identified yet, despite other PGPR strains for maize had been detected previously [35–37]. We explored the maize root endosphere microbiome and the effect of chilling temperatures on the root endosphere bacterial communities and assessed whether these results could facilitate the selection of PGPR strains to promote maize growth under chilling conditions.

By means of two different experimental setups, we identified 12 root endosphere families as the main microbiome of maize grown in field soil. We consider these families as robust colonizers over different experiments for the same soil sampled at various time points, confirming previous results in other crop systems [11–13]. Previously, a core maize microbiome has been determined at ASV level [38]. In total, eight of the 12 families we identified in our setup, had been described as core microbiome members [38]. Hence, these families, *Caulobacteraceae*, *Comamonadaceae*, *Cytophagaceae*, *Hyphomicrobiaceae*, *Oxalobacteraceae*, *Rhizobacteraceae*, *Streptomycetaceae*, and *Xanthomonadales* can be considered as true core microbiome families of maize. The differences however confirm that the root endosphere community is also highly influenced by factors, such as soil, genotype, experimental set-up, and climatic conditions [12, 13, 39, 40]**.**

Bacterial inoculants currently on the market are applied via seed coatings indicating that the bacterial inoculant should be able to colonize the maize roots from the surroundings [41, 42]. Hence, we hypothesized that seed-derived root endophytes would have a lower effcieny in root colonization than the bacteria that are recruited from the soil and thus are of less interest for application. Correspondingly, we did not screen these bacterial families for growth promotion. We revealed that the soil-grown root endosphere is only colonized to a minor extent by seed endophytes [43, 44]. Firstly, we validated that the family *Moraxellaceae* is recruited from the seed tissues because of its absence in the bulk soil samples and its abundance in the root endosphere of both soil- and in vitro-grown maize. Due to their absence in the bulk soil, we can speculate that members of the *Moraxellaceae* family are true endophytes that can be transmitted from the seeds to the root endosphere. This family has been previously discovered in root environments and several genera of the *Moraxellaceae* are known to promote plant growth [45–47]. Secondly, the family *Flavobacteriaceae* was detected in the root endosphere of both soil- and in vitro-grown maize and additionally, also in the bulk soil samples. Accordingly, we hypothesize that this family can colonize the root endosphere both through seed transmission and via the surrounding soil. Together, most of the detected root endophytes are recruited from the soil and can thus be used in seed coating strategies. Whether or not seed-transmitted bacteria, such as those belonging to the *Moraxellaceae,* perform worse in seed coating treatments than soil-recruited strains has to be tested in the future. Additionally, it would also be interesting to analyze whether recruitment of bacterial families from the seed into the root is influenced by environmental factors, such as chilling temperatures.

Abiotic stresses affect bacterial communities in root and soil [13, 18, 39]. A shift in the bacterial community was detected upon chilling conditions that, as under drought conditions [39], is more pronounced in the root endosphere than in the bulk soil. Hence, it is tempting to speculate that plants mediate the selection of the bacterial communities in the root endosphere under stress conditions. As changes in root exudates are known to affect the microbiome [18, 43, 48] and abiotic stress conditions to alter the root exudate composition [49, 50], the chilling effect on the root exudates might have an impact on the root endosphere microbiome. Therefore, it would be worthwhile to study whether the detected shifts are caused by the influence of temperature on the bacterial life cycle or on the entire bacterial population, by plant physiological alterations, or by a combination of these factors.

Based on two independent experiments under chilling stress, 12 chilling-responsive families were detected that were repeatedly enriched in the root endosphere upon chilling conditions. Additionally, three other chilling-responsive were recurrently depleted upon chilling temperatures of which the actinobacterial family *Streptomycetaceae* was the most striking one. This family has previously been shown to be enriched in endo- and rhizospheres of several grass species upon drought, indicating that not every abiotic stress provokes the same types of changes in the composition [19, 20]. Further experiments are needed to determine whether the decrease in *Streptomycetaceae* in our study is caused by the actinobacterial life cycle and whether their susceptibility to chilling conditions is indirectly affected by chilling on other bacterial families or by the alternate plant-mediated attraction of microbes during chilling periods.

We hypothesized that enriched chilling-responsive families might promote growth of maize under chilling conditions. To investigate whether the microbiome results could be used as a tool to facilitate PGPR selection, we screened a selection of maize endophytes from an established bacterial collection for their growth-promoting capacities under chilling conditions. RHG12 (*Oxalobacteraceae*–*Pseudoduganella* sp.) and RHG5 (*Bradyrhizobiaceae*–*Bosea* sp.) were identified as growth-promoting strains under chilling temperatures, whereas they did not under normal temperature conditions. Both belong to families that are, although known to be endophytic, not studied in-depth for these abilities. We demonstrated that *Oxalobacteraceae* are part of the main endosphere microbiome but not chilling responsive. As *Oxalobacteraceae* were not detected in the endosphere of in vitro-grown plants (experiment III), we hypothesize that this family colonized the root system from the soil environment. Further research on the maize–*Oxalobacteraceae* interaction and colonization will help to unravel questions regarding the underlying growth-promoting pathways and whether the plant has an active role in the recruitment of this bacterial family.

The *Bradyrhizobiaceae* family, to which RHG5 belongs, was enriched, however low abundant in the root endosphere. Similarly as for the *Oxalobacteraceae*, this family was not demonstrated to be chilling-responsive and not detected in the root endosphere of in vitro-grown plants. The family contains known rhizobia species that uniquely interact with legumes, such as *Bradyrhizobium japonicum* that nodulates soybean (*Glycine max*) [51]. Based on the genome, the family has been linked with nitrogen fixation [52], but the link with the detected growth promotion should however be further determined.

Thus, both identified PGPR strains belong to endosphere-enriched families of which neither were shown to be chilling-responsive, implying that strains do not necessarily have to be enriched in the endosphere upon chilling conditions to enhance plant growth under these conditions. At the sequence level, we only identified a matching ASV for RHG12, ID_10, which was highly abundant and strongly enriched in the root endosphere, whereas it did not shift under chilling conditions. Hence, enrichment and high abundance in the endosphere are not always required to promote plant growth. Robust root colonization on the contrary, typical for endophytes, might be required to maintain the growth-promoting effects on the plant, because it can indeed be assumed as a competitive advantage in the complex root-surrounding bacterial community [51, 53, 54]. Nevertheless, the effect of the identified strains has only been tested in the semi-sterile sand-perlite system and growth-promoting strains could have been missed because of the different environmental conditions. Ideally, validation should be done within field soil environments and preferentially in the field. Because such experiments are not feasible for large-scale screening assays, the effects of only the two identified PGPR should definitely be validated in the field in the future. Additionally, to better understand the link between root endosphere enrichment and PGPR, a larger screening with higher through-put should be pursued. More in-depth studies on the colonization of the different growth-promoting strains and their functional traits both in reductionist and complex approaches will further help to address this question.

## Conclusions

Plants create a stable root endosphere microbiome containing mostly bacteria that are attracted from the surrounding soil environment into the root endosphere, whereas only a few are derived from the seed. When grown under chilling conditions, the shift detected in the bacterial communities of the roots was more pronounced than that upon chilling in the bulk soil samples. Our screening attempts demonstrated that strains do not need to belong to families that respond to chilling temperatures to provoke growth promotion under these conditions. However, because both strains belong to root endosphere-enriched families and are expected to be good root colonizers, coating seeds with these strains might definitely help the growth of juvenile maize in chilling temperatures. Hence, the two PGPR strains identified here will be studied further in detail to explore their potential for agricultural applications.

## Supplementary information

**Additional file 1: Figure S1.** Difference in the relative abundances between experiments I and II of the phylum *Actinobacteria* and the *Streptomycetaceae* family. **Figure S2.** Shannon diversity indices (effective number of species) accounting for the within-sample diversity of the different sample types (all experiments) and between temperature conditions (experiments IV and V). **Figure S3.** PCoA plots of the endosphere and bulk soil samples of experiment IV and V. **Figure S4.** The effect of all the screened bacterial strains on the total fresh weight of maize grown under chilling conditions. **Figure S5.** The effect of RHG5 and RHG12 on maize grown under control temperature conditions. **Table S1.** Soil characteristics of the field soil used in the different experiments (determined by ILVO). **Table S2.** Composition of bacterial growth media used for isolation approaches. **Table S3.** Read counts of the different samples (before and after filtering) in each microbiome experiment. **Table S4.** Bacterial communities of bulk soil and endosphere in field and pot experiments I and II. **Table S5.** Bacterial seed endophytes (experiment III). **Table S6.** Results of the PERMANOVA analysis for experiments IV and V. **Table S7.** Relative abundance of bacterial communities of the temperature experiments IV and V. **Table S9.** Effect of the screened bacterial isolates on the root, shoot and total fresh weight. **Table S10.** Relative abundance of ASV ID_10 in experiment IV and V.

**Additional file 2:Table S8. A.** Summary of the phyla and families represented by the bacterial isolates in the bacterial collection. **Table S8 B.** Bacterial isolates of the collection with the assigned taxonomy. **Table S8 C.** Bacterial isolates of the collection with the 16S rRNA DNA sequence.

## Data Availability

The raw sequence reads were deposited and are available for download at the NCBI sequence reads archive (SRA) with project number PRJNA523518 (experiments I, II, and III) and PRJNA524079 (experiments IV and V).

## References

[1] Farooq M, Aziz T, Wahid A, Lee D-J, Siddique KHM (2009). Chilling tolerance in maize: agronomic and physiological approaches. Crop Pasture Sci..

[2] Leipner J, Stamp P, Fracheboud Y (2000). Artificially increased ascorbate content affects zeaxanthin formation but not thermal energy dissipation or degradation of antioxidants during cold-induced photooxidative stress in maize leaves. Planta..

[3] Leipner J, Stamp P, Bennetzen JL, Hake SC (2009). Chilling stress in maize seedlings. Handbook of Maize: Its Biology.

[4] Sobkowiak A, Jończyk M, Adamczyk J, Szczepanik J, Solecka D, Kuciara I (2016). Molecular foundations of chilling-tolerance of modern maize. BMC Genomics..

[5] Zhang R, Vivanco JM, Shen Q (2017). The unseen rhizosphere root–soil–microbe interactions for crop production. Curr Opin Microbiol..

[6] Berg G, Grube M, Schloter M, Smalla K (2014). Unraveling the plant microbiome: looking back and future perspectives. Front Microbiol..

[7] Backer R, Rokem JS, Ilangumaran G, Lamont J, Praslickova D, Ricci E (2018). Plant growth-promoting rhizobacteria: context, mechanisms of action, and roadmap to commercialization of biostimulants for sustainable agriculture. Front. Plant Sci..

[8] Xu L, Coleman-Derr D (2019). Causes and consequences of a conserved bacterial root microbiome response to drought stress. Curr. Opin Microbiol..

[9] Bulgarelli D, Rott M, Schlaeppi K, Ver Loren van Themaat E, Ahmadinejad N, Assenza F (2012). Revealing structure and assembly cues for Arabidopsis root-inhabiting bacterial microbiota. Nature..

[10] Lundberg DS, Lebeis SL, Paredes SH, Yourstone S, Gehring J, Malfatti S (2012). Defining the core *Arabidopsis thaliana* root microbiome. Nature..

[11] Peiffer JA, Spor A, Koren O, Jin Z, Tringe SG, Dangl JL (2013). Diversity and heritability of the maize rhizosphere microbiome under field condtions. Proc Natl Acad Sci USA..

[12] Edwards J, Johnson C, Santos-Medellin C, Lurie E, Podishetty NK, Bhatnagar S (2015). Structure, variation, and assembly of the root-associated microbiomes of rice. Proc Natl Acad Sci USA..

[13] Coleman-Derr D, Desgarennes D, Fonseca-Garcia C, Gross S, Clingenpeel S, Woyke T (2016). Plant compartment and biogeography affect microbiome composition in cultivated and native *Agave* species. New Phytol..

[14] Johnston-Monje D, Raizada MN (2011). Conservation and diversity of seed associated endophytes in *Zea* across boundaries of evolution, ethnography and ecology. PLoS ONE..

[15] Truyens S, Weyens N, Cuypers A, Vangronsveld J (2015). Bacterial seed endophytes: genera, vertical transmission and interaction with plants. Environ Microbiol Rep..

[16] Rahman MDM, Flory E, Koyro H-W, Abideen Z, Schikora A, Suarez C (2018). Consistent associations with beneficial bacteria in the seed endosphere of barley (*Hordeum vulgare* L.). Syst Appl Microbiol.

[17] Kwak M-J, Kong HG, Choi K, Kwon S-K, Song JY, Lee J (2018). Rhizosphere microbiome structure alters to enable wilt resistance in tomato. Nat Biotechnol..

[18] Carríon VJ, Perez-Jaramillo J, Cordovez V, Tracanna V, de Hollander M, Ruiz-Buck D (2019). Pathogen-induced activation of disease-suppressive functions in the endophytic root microbiome. Science.

[19] Naylor D, Coleman-Derr D (2018). Drought stress and root-associated bacterial communities. Front Plant Sci..

[20] Yuan Z, Druzhinina IS, Labbé J, Redman R, Qin Y, Rodriguez R (2016). Specialized microbiome of a halophyte and its role in helping non-host plants to withstand salinity. Sci. Rep..

[21] Theocharis A, Bordiec S, Fernandez O, Paquis S, Dhondt-Cordelier S, Baillieul F (2012). *Burkholderia phytofirmans* PsJN primes *Vitis vinifera* L. and confers a better tolerance to low nonfreezing temperatures. Mol Plant-Microbe Interact..

[22] Su F, Jacquard C, Villaume S, Michel J, Rabenoelina F, Clément C (2015). *Burkholderia phytofirmans PsJN* reduces impact of freezing temperatures on photosynthesis in *Arabidopsis thaliana*. Front Plant Sci..

[23] Tiwari S, Prasad V, Chauhan PS, Lata C (2017). *Bacillus amyloliquefaciens* confers tolerance to various abiotic stresses and modulates plant response to phytohormones through osmoprotection and gene expression regulation in rice. Front. Plant Sci..

[24] Mishra PK, Bisht SC, Ruwari P, Selvakumar G, Joshi GK, Bisht JK (2011). Alleviation of cold stress in inoculated wheat (*Triticum aestivum* L.) seedlings with psychrotolerant Pseudomonads from NW Himalayas. Arch. Microbiol.

[25] Kakar KU, Ren X-L, Nawaz Z, Cui Z-Q, Li B, Xie G-L (2016). A consortium of rhizobacterial strains and biochemical growth elicitors improve cold and drought stress tolerance in rice (*Oryza sativa* L.). Plant Biol..

[26] Subramanian P, Kim K, Krishnamoorthy R, Mageswari A, Selvakumar G, Sa T (2016). Cold stress tolerance in psychrotolerant soil bacteria and their conferred chilling resistance in tomato (*Solanum lycopersicum* Mill.) under low temperatures. PLoS ONE.

[27] Andrews S (2010). FastQC: a quality control tool for high throughput sequence data.

[28] Callahan BJ, McMurdie PJ, Rosen MJ, Han AW, Johnson AJA, Holmes SP (2016). DADA2: high-resolution sample inference from Illumina amplicon data. Nat Methods..

[29] Quast C, Pruesse E, Yilmaz P, Gerken J, Schweer T, Yarza P (2013). The SILVA ribosomal RNA gene database project: improved data processing and web-based tools. Nucleic Acids Res..

[30] Niemann S, Pühler A, Tichy H-V, Simon R, Selbitschka W (1997). Evaluation of the resolving power of three different DNA fingerprinting methods to discriminate among isolates of a natural *Rhizobium meliloti* population. J Appl Microbiol..

[31] Oksanen J, Blanchet FG, Kindt R, Legendre P, Minchin P, O’Hara RB, Simpson G, et al. Vegan: Community Ecology Package. R Package Version. 2.0-10. The Comprehensive R Archive Network (CRAN) 2019. https://cran.r-project.org/.

[32] Robinson MD, McCarthy DJ, Smyth GK (2010). edgeR: a Bioconductor package for differential expression analysis of digital gene expression data. Bioinformatics..

[33] Johnston-Monje D, Mousa WK, Lazarovits G, Raizada MN (2014). Impact of swapping soils on the endophytic bacterial communities of pre-domesticated, ancient and modern maize. BMC Plant Biol..

[34] Barret M, Briand M, Bonneau S, Préveaux A, Valière S, Bouchez O (2015). Emergence shapes the structure of the seed microbiota. Appl Environ Microbiol..

[35] Naveed M, Mitter B, Reichenauer TG, Wieczorek K, Sessitsch A (2014). Increased drought stress resilience of maize through endophytic colonization by *Burkholderia phytofirmans* PsJN and *Enterobacter* sp. FD17. Environ Exp Bot..

[36] Li Y, Liu X, Hao T, Chen S (2017). Colonization and maize growth promotion induced by phosphate solubilizing bacterial isolates. Int J Mol Sci..

[37] Oliveira ALM, Santos OJAP, Marcelino PRF, Milani KML, Zuluaga MYA, Zucareli C (2017). Maize inoculation with *Azospirillum brasilense* Ab-V5 cells enriched with exopolysaccharides and polyhydroxybutyrate results in high productivity under low N fertilizer input. Front Microbiol..

[38] Walters WA, Jin Z, Youngblut N, Wallace JG, Sutter J, Zhang W (2018). Large-scale replicated field study of maize rhizosphere identifies heritable microbes. Proc Natl Acad Sci USA..

[39] Naylor D, DeGraaf S, Purdom E, Coleman-Derr D (2017). Drought and host selection influence bacterial community dynamics in the grass root microbiome. ISME J..

[40] Fitzpatrick CR, Copeland J, Wang PW, Guttman DS, Kotanen PM, Johnson MTJ (2018). Assembly and ecological function of the root microbiome across angiosperm plant species. Proc. Natl. Acad. Sci. USA.

[41] Sergaki C, Lagunas B, Lidbury I, Gifford ML, Schäfer P (2018). Challenges and approaches in microbiome research: from fundamental to applied. Front Plant Sci..

[42] Sessitsch A, Brader G, Pfaffenbichler N, Gusenbauer D, Mitter B (2018). The contribution of plant microbiota to economy growth. Microb Biotechnol..

[43] Berg G, Raaijmakers JM (2018). Saving seed microbiomes. ISME J..

[44] Shahzad R, Khan AL, Bilal S, Asaf S, Lee IJ (2018). What is there in seeds? Vertically transmitted endophytic resources for sustainable improvement in plant growth. Front Plant Sci.

[45] Panke-Buisse K, Poole AC, Goodrich JK, Ley RE, Kao-Kniffin J (2015). Selection on soil microbiomes reveals reproducible impacts on plant function. ISME J..

[46] Benitez M-S, Osborne SL, Lehman RM (2017). Previous crop and rotation history effects on maize seedling health and associated rhizosphere microbiome. Sci Rep..

[47] Armanhi JSL, de Souza RSC, Damasceno ND, de Araujo LM, Imperial J, Arruda P (2018). A community-based culture collection for targeting novel plant growth-promoting bacteria from the sugarcane microbiome. Front Plant Sci..

[48] Kudjordjie EN, Sapkota R, Steffensen SK, Fomsgaard IS, Nicolaisen M (2019). Maize synthesized benzoxazinoids affect the host associated microbiome. Microbiome.

[49] Liu Y, Mi GH, Chen FJ, Zhang JH, Zhang FS (2004). Rhizosphere effect and root growth of two maize (*Zea mays* L.) genotypes with contrasting P efficiency at low P availability. Plant Sci..

[50] Javed MT, Akram MS, Tanwir K, Chaudhary HJ, Ali Q, Stoltz E (2017). Cadmium spiked soil modulates root organic acids exudation and ionic contents of two differentially Cd tolerant maize (*Zea mays* L.) cultivars. Ecotox Environ Safe.

[51] Itakura M, Saeki K, Omori H, Yokoyama T, Kaneko T, Tabata S (2009). Genomic comparison of *Bradyrhizobium japonicum* strains with different symbiotic nitrogen-fixing capabilities and other Bradyrhizobiaceae members. ISME J..

[52] De Meyer SE, Willems A. Multilocus sequence analysis of Bosea species and description of Bosea lupini sp. nov., Bosea lathyri sp. nov. and Bosea robiniae sp. nov., isolated from legumes. Int J Syst Evol Microbiol.2012;62:2505-10. 10.1099/ijs.0.035477-0 22155761

[53] Lebeis SL, Paredes SH, Lundberg DS, Breakfield N, Gehring J, McDonald M (2015). Salicylic acid modulates colonization of the root microbiome by specific bacterial taxa. Science..

[54] van der Meij A, Willemse J, Schneijderberg MA, Geurts R, Raaijmakers JM, van Wezel GP (2018). Inter- and intracellular colonization of *Arabidopsis* roots by endophytic actinobacteria and the impact of plant hormones on their antimicrobial activity. Antonie Van Leeuwenhoek..

