# Supplementary material for: Tapping into the maize root microbiome to identify bacteria that promote growth under chilling conditions
Source: Microbiome. 2020 Apr 18;8:54. doi: 10.1186/s40168-020-00833-w (PMC7166315; doi:10.1186/s40168-020-00833-w)
Supplement: Supplementary file 2 — Additional file 1: Figure S1. Difference in the relative abundances between experiments I and II of the phylum Actinobacteria and the Streptomycetaceae family. Figure S2. Shannon diversity indices (effective number of species) accounting for the within-sample diversity of the different sample types (all experiments) and between temperature conditions (experiments IV and V). Figure S3. PCoA plots of the endosphere and bulk soil samples of experiment IV and V. Figure S4. The effect of all the screened bacterial strains on the total fresh weight of maize grown under chilling conditions. Figure S5. The effect of RHG5 and RHG12 on maize grown under control temperature conditions. Table S1. Soil characteristics of the field soil used in the different experiments (determined by ILVO). Table S2. Composition of bacterial growth media used for isolation approaches. Table S3. Read counts of the different samples (before and after filtering) in each microbiome experiment. Table S4. Bacterial communities of bulk soil and endosphere in field and pot experiments I and II. Table S5. Bacterial seed endophytes (experiment III). Table S6. Results of the PERMANOVA analysis for experiments IV and V. Table S7. Relative abundance of bacterial communities of the temperature experiments IV and V. Table S9. Effect of the screened bacterial isolates on the root, shoot and total fresh weight. Table S10. Relative abundance of ASV ID_10 in experiment IV and V. [file 40168_2020_833_MOESM1_ESM.pdf]

SUPPORTING INFORMATION

Fig. S1. Differences in the relative abundances between experiments I and II of the phylum *Actinobacteria* and the family *Streptomycetaceae*. The relative abundance (%) of the *Actinobacteria* ASVs that are assigned to the *Streptomycetaceae* family in both experiments are shown in the bottom panel.

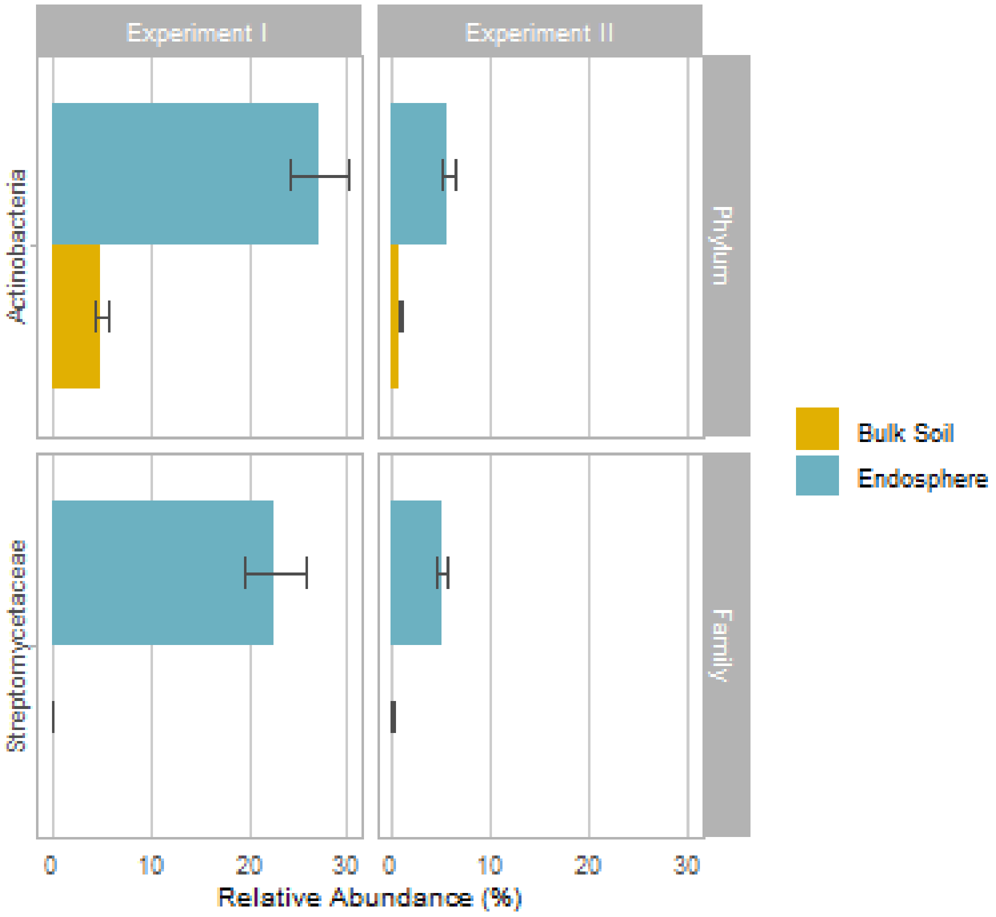

Fig. S2. Shannon diversity indices (effective number of species) accounting for the within-sample diversity of the different sample types (all experiments) and between temperature conditions (experiments IV and V).

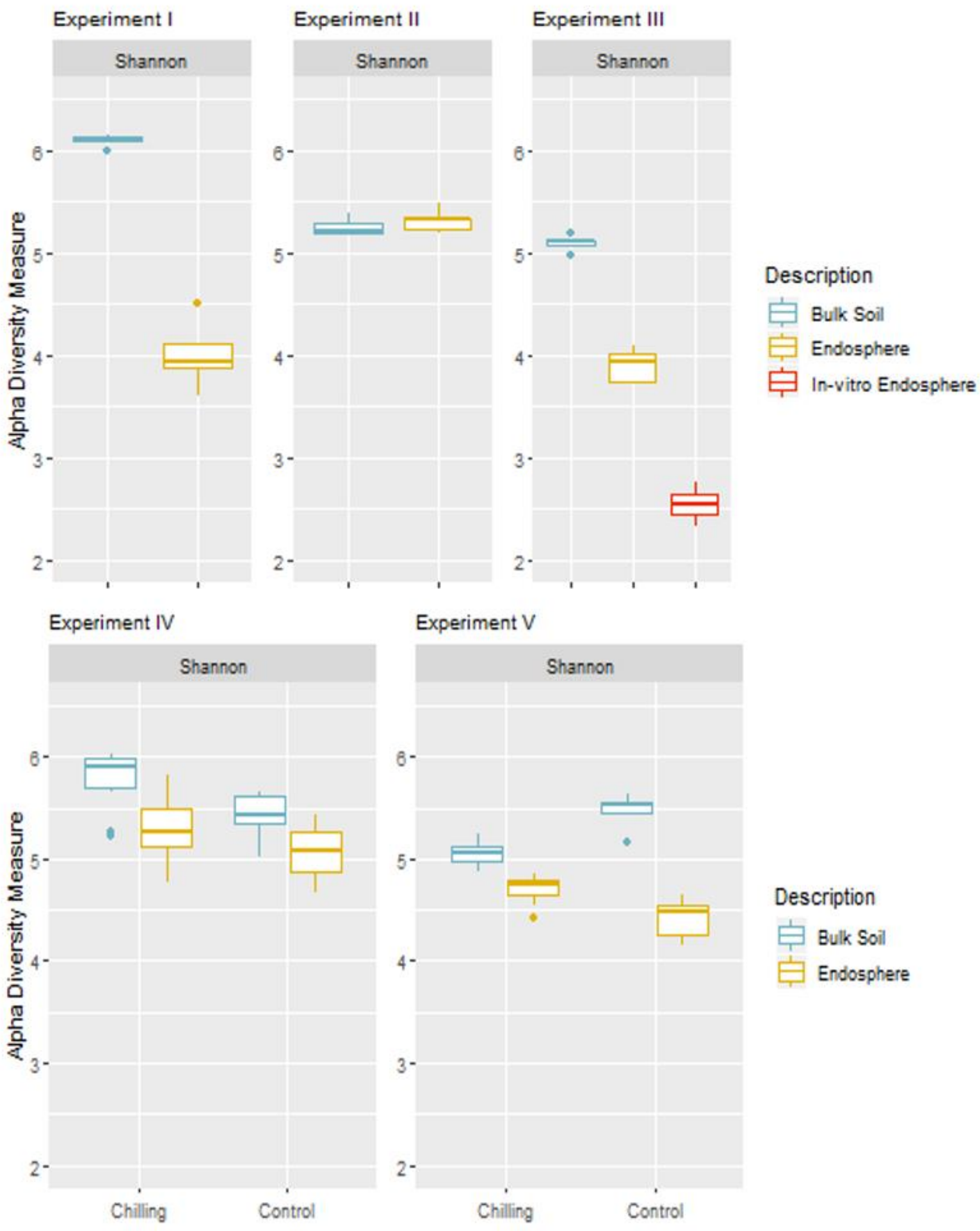

Fig. S3. Principal coordinate analysis of the microbial communities of bulk soil and endosphere in both chilling and control temperatures (experiments IV and V). PCoA plots are based on Bray-Curtis dissimilarity indices.

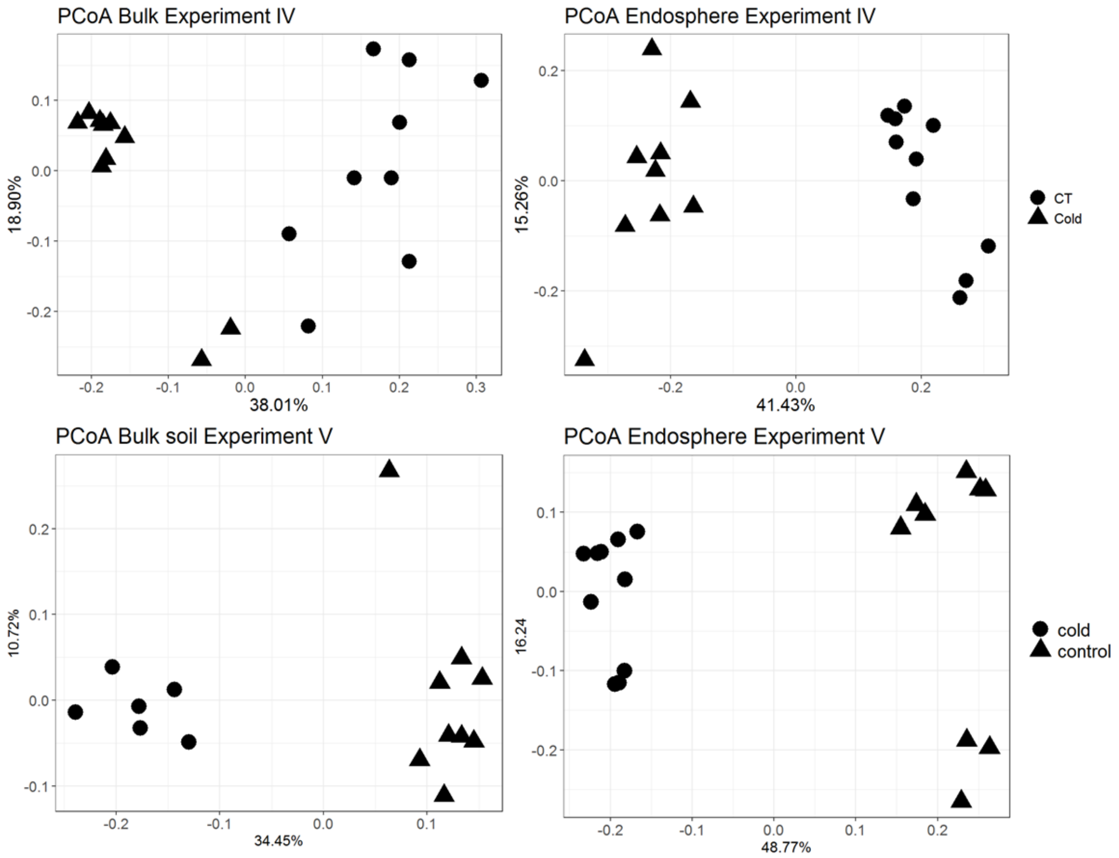

Fig. S4. Screening of isolates of the bacterial collection. In total, 28 different bacterial strains were screened for their effect on juvenile maize growth under chilling temperature conditions. The effect of the bacterial isolates on the total (shoot and root) fresh weight (in g) was tested in different repeats. The white and grey boxes represent the mock-inoculated and bacteria-inoculated plants, respectively. Individual datapoints are presented as black dots. For the statistical analysis and taxonomy of the bacterial isolates , see Table S9.

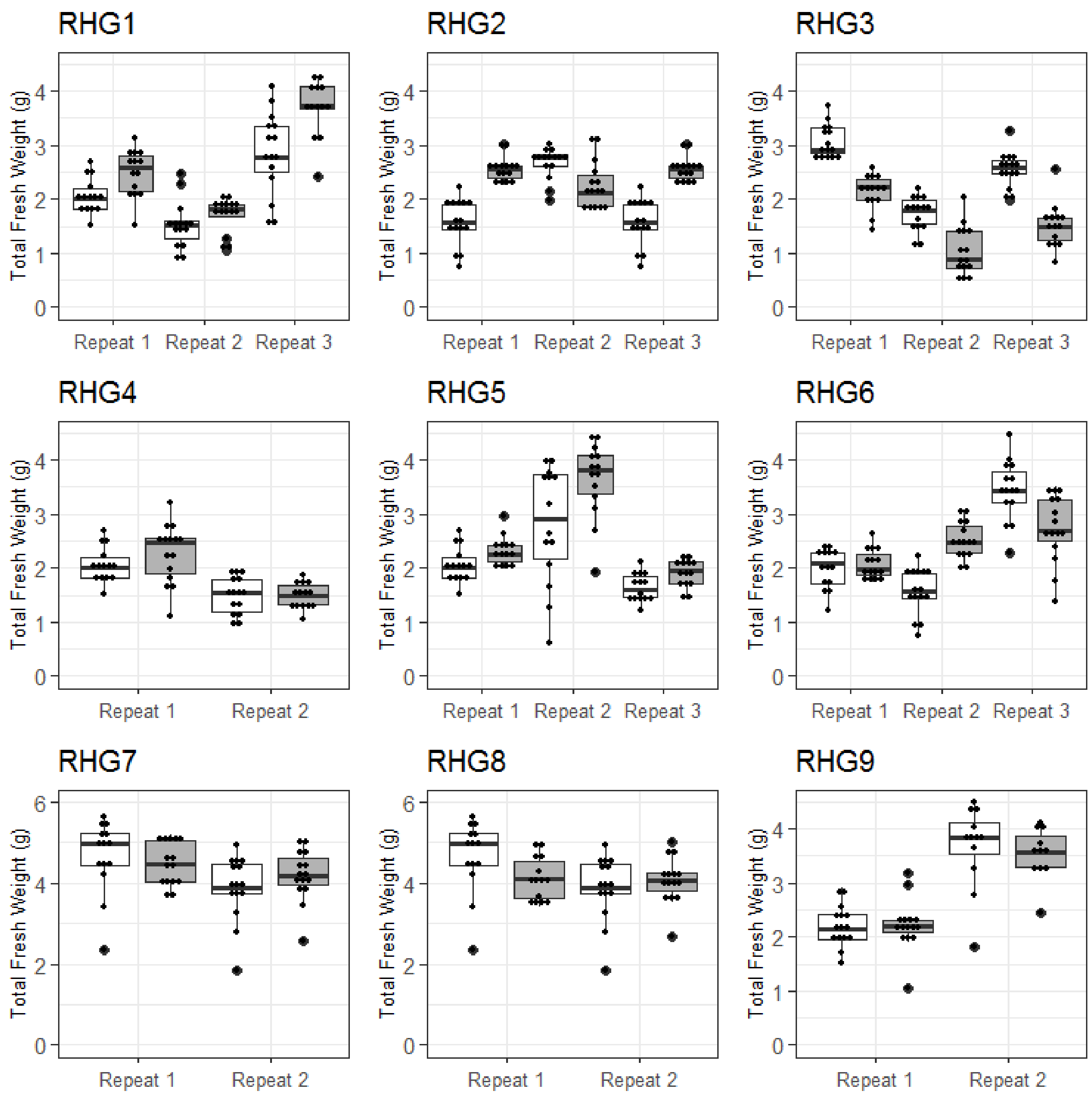

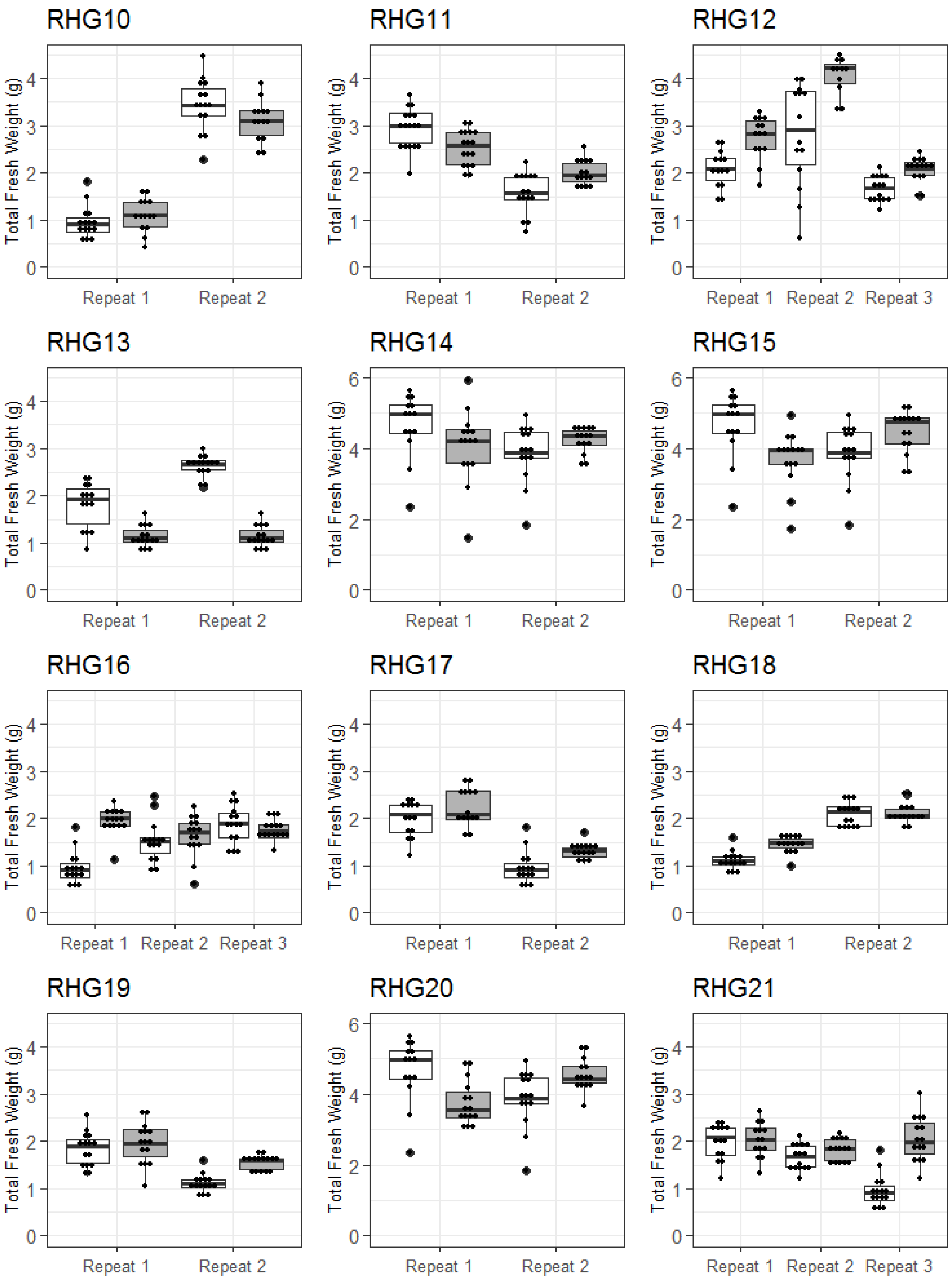

RHG10
RHG11
RHG12
RHG13
RHG14
RHG15
RHG16
RHG17
RHG18
RHG19
RHG20
RHG21
Total Fresh Weight (g)
Repeat 1
Repeat 2
Repeat 3

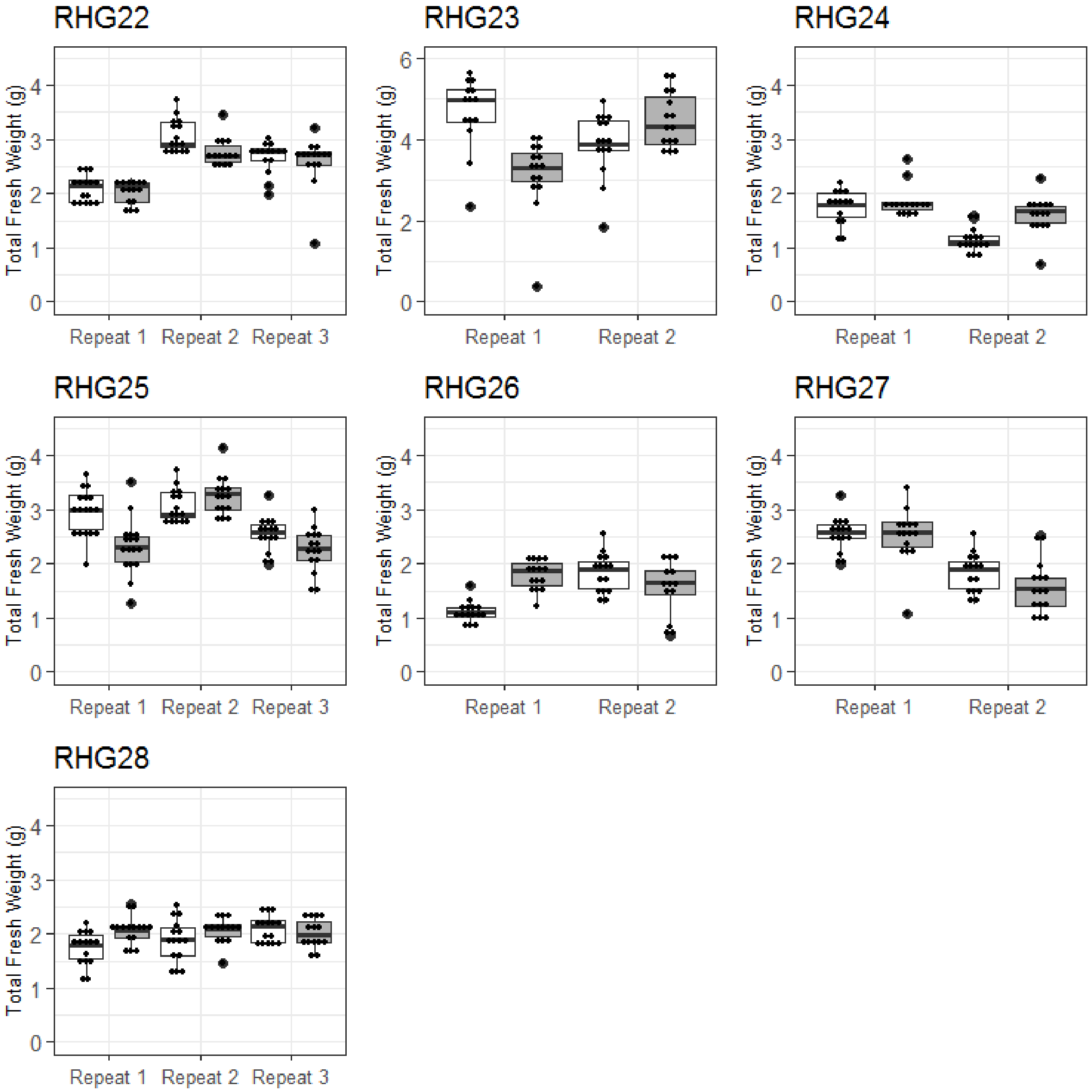
RHG22
RHG23
RHG24
RHG25
RHG26
RHG27
RHG28
Total Fresh Weight (g)
Repeat 1
Repeat 2
Repeat 3
0
1
2
3
4
6

Fig. S5. Effect of RHG5 and RHG12 on juvenile maize growth under normal temperature conditions. The two identified PGPR strains, *Bosea* RHG5 and *Pseudoduganella* RHG12, were used to inoculate maize seedlings and their effect on the total (shoot and root) fresh weight was analyzed when maize was grown under control temperature conditions. Total fresh weight of bacterial and mock inoculated (n = 15) plants were measured and compared in three repeats. A two-sample Student's t-test was used to detect significant effects of the inoculation. The white box represents the control condition, whereas the grey box represents the treated conditions. Individual datapoints are shown as black dots.

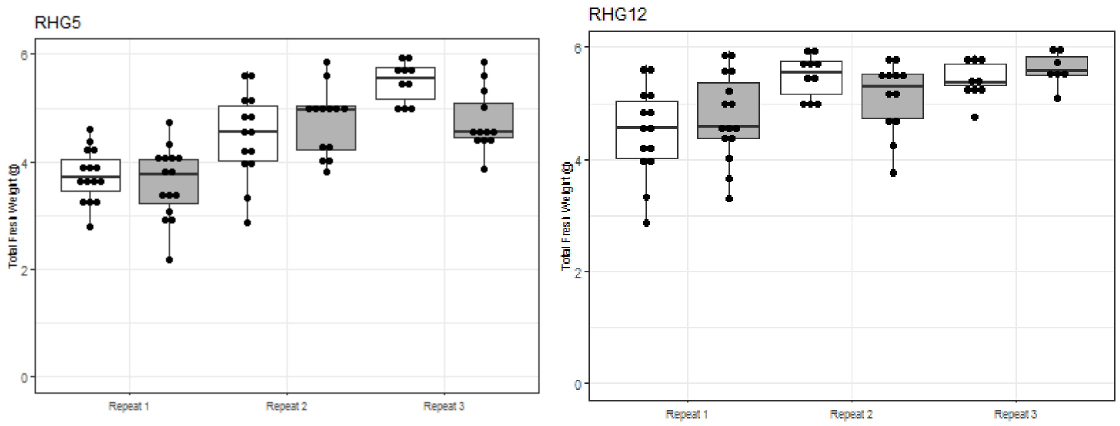

Table S1. Soil characteristics of the field soil used in the different experiments (determined by ILVO).

**Analysis**	**Merelbeke soil**
N totaal	0.11% dry matter (DM)
Fe-AmLact	45.5 mg/100 g DM
K-AmLact	20.2 mg/100 g DM
DS	88.1% fresh matter
OC	1.12% DM
pH KCl	6.16
Mg-AmLact	21.6 mg/100 g DM
Ca-AmLact	114.4 mg/100 g DM
Mn-AmLact	13 mg/100 g DM
Na-AmLact	< 1.92 mg/100 g DM
P-AmLact	22 mg/100 g DM

Table S2. Composition of bacterial growth media used for isolation approaches

Media	Composition
**For plating**	15 g/l agar (Sigma-Aldrich)
**R2A** (pH 7)	0.5 g/l Proteose peptone (Sigma-Aldrich)
	0.5 g/l Casamino acids (Thermo Fisher)
	0.5 g/l Yeast extract (Sigma-Aldrich)
	0.5 g/l Dextrose (Sigma-Aldrich)
	0.5 g/l Soluble starch (Thermo Fisher)
	0.3 g/l K_2HPO_4 (Sigma-Aldrich)
	0.5 g/l $MgSO_4$ (Sigma-Aldrich)
	0.3 g/l Sodium pyruvate (Thermo Fisher)
**TSB** (pH 7.3)	17 g/l Pancreatic Digest of Casein (Sigma-Aldrich)
	3 g/l Papaic Digest of Soybean (Sigma-Aldrich)
	5 g/l NaCl (Chem lab)
	2.5 g/l K_2HPO_4 (Sigma-Aldrich)
	2.5 g/l Dextrose (Sigma-Aldrich)
**King's B** (pH 7)	15 g proteose peptone #2 (DIFCO)
	1.5 g/l K_2HPO_4 (Sigma-Aldrich)
	5 ml $MgSO_4$ (1 M; sterile)

Table S3. Read counts of the different samples (before and after filtering) in each microbiome experiment. During filtering, chloroplasts and mitochondria reads were removed, together with reads belonging to low abundant ASVs (< 2 counts/ASV in at least five samples)

Experiment	Samples	Reads per sample	Reads per sample after filtering	ASVs per sample
I	Bulk soil	120,092	95,139	925
	Root endosphere	310,122	62,591	466
II	Bulk soil	58,412	57,960	685
	Root endosphere	286,594	94,250	608
III	Bulk soil	57,129	56,717	426
	Root endosphere	233,639	24,731	219
	In vitro	235,120	1,920	21
IV	Bulk control	23,814	23,579	814
	Bulk chilling	44,789	44,694	544
	Root endosphere control	136,529	35,846	878
	Root endosphere chilling	102,533	20,544	584
V	Bulk control	19,871	19,786	636
	Bulk chilling	16,274	16,220	461
	Root endosphere control	92,465	30,933	530
	Root endosphere chilling	124,026	38,947	497

Table S4. Bacterial communities of bulk soil and root endosphere in field- and pot-grown maize of experiments I and II. Relative abundance ± SE (%) are shown; Phyla are highlighted. Asterisks represent *P* values of significant difference in the root endosphere compared to bulk soil: * *P* <0.05, ** *P* <0.001; *** *P* <0.001.

**Family**	**Experiment I**		**Experiment II**	
	Bulk	Endosphere	Bulk	Endosphere
Proteobacteria	26.78 ± 0.70	52.42 ± 2.14***	21.69 ± 0.45	46.09 ± 3.52***
Oxalobacteraceae	2.61 ± 0.76	27.77 ± 3.16***	0.14 ± 0.01	3.62 ± 0.35***
Comamonadaceae	0.91 ± 0.13	9.01 ± 1.46***	0.36 ± 0.09	8.66 ± 0.95***
Rhizobiaceae	0.02 ± 0.01	0.84 ± 0.14***	0.00 ± 0.00	1.25 ± 0.21***
Caulobacteraceae	0.16 ± 0.03	0.64 ± 0.06***	0.01 ± 0.01	1.77 ± 0.26***
BIrii41	0.24 ± 0.04	3.10 ± 0.65***	0.89 ± 0.14	3.24 ± 0.32***
Cellvibrionaceae	0.01 ± 0.01	0.74 ± 0.19***	-	-
Bradyrhizobiaceae	0.31 ± 0.04	0.85 ± 0.12***	0.33 ± 0.11	1.02 ± 0.10
Hyphomicrobiaceae	0.59 ± 0.14	1.41 ± 0.16***	0.48 ± 0.05	1.74 ± 0.05***
Haliangiaceae	0.50 ± 0.05	0.64 ± 0.07***	0.70 ± 0.15	0.52 ± 0.08
Sphingomonadaceae	1.61 ± 0.27	0.64 ± 0.05	0.47 ± 0.08	1.38 ± 0.29***
Xanthomonadales_Incertae_Sedis	0.91 ± 0.05	1.11 ± 0.26***	1.45 ± 0.17	3.97 ± 0.39***
Xanthomonadaceae	2.59 ± 0.45	1.37 ± 0.15	1.03 ± 0.11	5.31 ± 1.68***
Polyangiaceae	0.00 ± 0.00	1.17 ± 0.17***	0.19 ± 0.06	0.27 ± 0.05
Enterobacteriaceae	0.04 ± 0.01	0.39 ± 0.07***	0.00 ± 0.00	5.59 ± 3.77***
Oceanospirillaceae	0.01 ± 0.01	0.01 ± 0.01	0.08 ± 0.02	0.56 ± 0.11***
Pseudomonadaceae	0.15 ± 0.04	0.27 ± 0.06**	0.20 ± 0.04	0.63 ± 0.16***
Rhodospirillaceae	0.53 ± 0.04	0.26 ± 0.02	0.11 ± 0.02	0.74 ± 0.09***
Actinobacteria	4.95 ± 0.72	27.19 ± 2.92***	0.77 ± 0.12	5.68 ± 0.64***
Streptomycetaceae	0.06 ± 0.01	22.67 ± 3.18***	0.04 ± 0.02	5.08 ± 0.58***
Thermomonosporaceae	0.01 ± 0.01	1.47 ± 0.07***	0.01 ± 0.01	0.34 ± 0.03
Pseudonocardiaceae	0.02 ± 0.02	1.20 ± 0.21***	-	-
Bacteroidetes	9.49 ± 0.94	13.17 ± 1.50***	16.14 ± 1.20	23.67 ± 2.15**
Cytophagaceae	2.74 ± 0.44	6.87 ± 1.01***	1.92 ± 0.17	10.49 ± 1.24***
Flavobacteriaceae	0.32 ± 0.13	1.33 ± 0.11**	0.49 ± 0.09	1.23 ± 0.70*
Chitinophagaceae	4.41 ± 0.40	4.16 ± 0.49*	6.80 ± 0.50	6.82 ± 0.45
Sphingobacteriaceae	0.6	0.36	0.14 ± 0.04	2.82 ± 1.20***
Cryomorphaceae	0.03 ± 0.01	0.09 ± 0.02***	0.12 ± 0.03	0.66 ± 0.09***
Chloroflexi	3.74 ± 0.27	2.45 ± 0.56***	3.53 ± 0.29	6.11 ± 0.87***
Anaerolineaceae	0.79 ± 0.07	1.53 ± 0.39***	2.08 ± 0.26	5.85 ± 0.84***
Firmicutes	1.54 ± 0.26	1.65 ± 0.23***	1.63 ± 0.23	2.59 ± 0.67*
Paenibacillaceae	0.06 ± 0.02	0.83 ± 0.23***	0.01 ± 0.01	0.01 ± 0.01
Erysipelotrichaceae	0.00 ± 0.00	0.66 ± 0.08***	0.00 ± 0.00	2.40 ± 0.70***
Fibrobacteres	0.00 ± 0.00	0.08 ± 0.03***	0.05 ± 0.02	0.45 ± 0.03***
Verrucomicrobia	5.60 ± 0.43	1.20 ± 0.16	11.55 ± 0.71	6.20 ± 0.69*
Opitutaceae	0.29 ± 0.05	0.38 ± 0.07**	0.69 ± 0.10	1.60 ± 0.24***
Verrucomicrobiaceae	0.24 ± 0.05	0.24 ± 0.06	0.23 ± 0.04	2.33 ± 0.30***
Acidobacteria	28.77 ± 0.70	1.34 ± 0.13***	29.26 ± 0.86	3.19 ± 0.47*
Spirochaetae	-	-	0.17 ± 0.03	1.62 ± 0.34***
Leptospiraceae	-	-	0.12 ± 0.03	0.73 ± 0.09**
Spirochaetaceae	-	-	0.05 ± 0.02	0.89 ± 0.33**

Table S5. Bacterial seed endophytes (experiment III)**.** ASVs of the root endosphere of the *in vitro*-grown plants, assigned taxonomy of each ASV, and the mean relative abundance in the bulk soil, the root endosphere of the soil-grown plants and the *in vitro*-grown plants (in % ± SE)

**Phylum**	**Family**	**Genus**	**ASV_ID**	**Bulk soil**	**Endosphere of soil-grown plants**	**Endosphere *in vitro*-grown plants**
Bacteroidetes	*Flavobacteriaceae*	*Chryseobacterium*	ID_108	0.00 ± 0.00	0.18 ± 0.02	13.06 ± 0.59
		Flavobacterium	ID_796	0.00 ± 0.00	0.00 ± 0.00	3.16 ± 0.62
	Sphingobacteriaceae	*Sphingobacterium*	ID_282	0.00 ± 0.00	0.02 ± 0.01	2.07 ± 0.26
			ID_696	0.00 ± 0.00	0.01 ± 0.01	1.41 ± 0.48
Firmicutes	*Paenibacillaceae*	*Paenibacillus*	ID_291	0.00 ± 0.00	0.00 ± 0.00	3.68 ± 0.34
Proteobacteria	*Rhizobiaceae*	*Shinella*	ID_102	0.00 ± 0.00	0.00 ± 0.00	2.69 ± 0.67
	Phyllobacteriaceae	*Phyllobacterium*	ID_1015	0.00 ± 0.00	0.00 ± 0.00	1.83 ± 0.64
	Brucellaceae	*Brucella*	ID_685	0.00 ± 0.00	0.00 ± 0.00	1.63 ± 0.55
	Oxalobacteraceae	*Pseudoduganella*	ID_668	0.00 ± 0.00	0.00 ± 0.00	2.45 ± 0.56
	Comamonadaceae	*Curvibacter*	ID_618	0.00 ± 0.00	0.00 ± 0.00	2.08 ± 0.69
	Enterobacteriaceae	NA	ID_4	0.07 ± 0.05	0.86 ± 0.15	8.69 ± 0.80
	Moraxellaceae	*Psychrobacter*	ID_84	0.00 ± 0.00	0.19 ± 0.03	16.73 ± 1.00
		Acinetobacter	ID_142	0.00 ± 0.00	0.16 ± 0.04	11.98 ± 0.62
			ID_228	0.00 ± 0.00	0.04 ± 0.04	9.23 ± 1.20
			ID_261	0.00 ± 0.00	0.00 ± 0.00	4.51 ± 1.23
			ID_659	0.00 ± 0.00	0.00 ± 0.00	2.75 ± 1.26
			ID_738	0.00 ± 0.00	0.00 ± 0.00	1.83 ± 0.64
	Pseudomonadaceae	*Pseudomonas*	ID_286	0.00 ± 0.00	0.00 ± 0.00	4.86 ± 1.37
			ID_66	0.03 ± 0.03	0.01 ± 0.01	3.27 ± 1.50
			ID_371	0.00 ± 0.00	0.34 ± 0.14	0.21 ± 0.21
	Xanthomonadaceae	*Stenotrophomonas*	ID_759	0.00 ± 0.00	0.00 ± 0.00	1.89 ± 0.87

Table S6. Results of the PERMANOVA test on the bulk soil and root endosphere samples of experiments IV and V. A PERMANOVA analysis was carried out on the Bray-Curtis dissimilarity matrices of the separate compartments (bulk soil or root endosphere) of both experiments IV and V. Results are listed in the table (vegan::Adonis(dissimilarity_matrix ~ Temperature))

**Compartment**	**R^2**	***P*-value**
Experiment IV		
Bulk Soil	0.33675	0.001
Endosphere	0.39649	0.001
Experiment V		
Bulk Soil	0.33238	0.001
Endosphere	0.47981	0.001

Table S7. Relative abundance of bacterial communities of the temperature experiments IV and V**.** Most abundant families and phyla in the root endosphere of maize grown under control and chilling temperature conditions, analysis was performed both at phylum and at family level. Asterisks represent *P* values of significant enrichment in the root endosphere under chilling conditions compared to the control conditions: * *P* <0.05, ** *P* <0.001; *** *P* <0.001

Family	Bulk		Endosphere	
	Control	Cold	Control	Cold
**Experiment IV**				
Proteobacteria	21.64 ± 1.34	24.18 ± 0.61	49.41 ± 1.56	71.48 ± 2.56
Oxalobacteraceae	0.64 ± 0.49	1.24 ± 0.18	18.33 ± 2.64	23.81 ± 3.20
Comamonadaceae	1.00 ± 0.16	1.34 ± 0.18	11.09 ± 0.95	20.70 ± 1.69
Rhizobiaceae	0.01 ± 0.01	0.03 ± 0.01	1.66 ± 0.31	1.01 ± 0.14
Caulobacteraceae	0.01 ± 0.01	0.16 ± 0.04	1.20 ± 0.17	1.43 ± 0.34
BIrii41	0.46 ± 0.08	0.85 ± 0.08	3.94 ± 0.46	3.66 ± 0.50
Bradyrhizobiaceae	0.79 ± 0.17	0.48 ± 0.07	1.61 ± 0.13	1.24 ± 0.10
Sphingomonadaceae	0.26 ± 0.10	0.16 ± 0.04	1.88 ± 0.32	2.41 ± 0.40
Xanthomonadaceae	0.84 ± 0.16	1.52 ± 0.12	1.86 ± 0.57	2.64 ± 0.71
Enterobacteriaceae	0.32 ± 0.32	0.00 ± 0.00	0.23 ± 0.08	3.56 ± 1.31
Pseudomonadaceae	0.37 ± 0.17	0.32 ± 0.03	0.81 ± 0.26	3.12 ± 1.21***
Polyangiaceae	0.08 ± 0.03	0.00 ± 0.00	0.26 ± 0.09	0.88 ± 0.15
Methylophilaceae	0.10 ± 0.05	0.07 ± 0.02	0.73 ± 0.18	1.62 ± 0.31
Phyllobacteriaceae	0.09 ± 0.05	0.06 ± 0.02	0.88 ± 0.11	1.21 ± 0.23
Actinobacteria	5.67 ± 0.82	3.34 ± 0.20	29.08 ± 1.81	8.85 ± 1.73***
Streptomycetaceae	0.07 ± 0.05	0.05 ± 0.03	25.08 ± 1.52	6.36 ± 1.38
Pseudonocardiaceae	0.08 ± 0.05	0.00 ± 0.00	0.93 ± 0.23	0.60 ± 0.29
Bacteroidetes	6.98 ± 0.77	13.59 ± 1.31	13.17 ± 2.15	11.51 ± 1.12
Cytophagaceae	2.95 ± 0.50	4.04 ± 0.32	5.91 ± 0.95	5.10 ± 0.73
Flavobacteriaceae	0.44 ± 0.10	1.34 ± 0.15	0.42 ± 0.15	0.75 ± 0.19
Chitinophagaceae	1.69 ± 0.19	4.46 ± 0.52	5.57 ± 1.04	2.49 ± 0.46***
Sphingobacteriaceae	0.13 ± 0.09	0.51 ± 0.06	1.04 ± 0.37	2.91 ± 0.66
Verrucomicrobia	13.07 ± 0.99	7.39 ± 0.40	2.30 ± 0.27	3.02 ± 0.53
Opitutaceae	0.39 ± 0.08	0.53 ± 0.07	0.77 ± 0.11	0.99 ± 0.17
Burkholderiaceae	0.02 ± 0.02	0.00 ± 0.00	0.75 ± 0.34	0.71 ± 0.29
Cellvibrionaceae	0.05 ± 0.04	0.29 ± 0.05	0.06 ± 0.02	0.67 ± 0.19**
Chloroflexi	6.34 ± 0.45	4.18 ± 0.13	2.35 ± 0.29	1.31 ± 0.30
Anaerolineaceae	2.67 ± 0.40	1.43 ± 0.06	1.59 ± 0.25	0.63 ± 0.18***
Acidobacteria	27.02 ± 2.65	30.87 ± 2.09	1.90 ± 0.23	2.02 ± 0.46
Blastocatellaceae	10.11 ± 1.79	14.07 ± 1.10	0.49 ± 0.05	0.61 ± 0.17
Thaumarchaeota	0.96 ± 0.13	0.52 ± 0.03	0.26 ± 0.03	0.40 ± 0.06**
Firmicutes	0.68 ± 0.15	0.67 ± 0.08	0.61 ± 0.14	0.33 ± 0.11
Planctomycetes	5.02 ± 0.29	2.57 ± 0.20	0.45 ± 0.13	0.29 ± 0.09
**Experiment V**				
Proteobacteria	30.81 ± 0.51	18.33 ± 0.67	72.73 ± 1.28	77.41 ± 1.06***
Oxalobacteraceae	1.61 ± 0.17	1.01 ± 0.09	34.17 ± 2.47	21.84 ± 1.39***
Comamonadaceae	2.39 ± 0.16	0.95 ± 0.06	13.37 ± 1.40	16.83 ± 0.68
Rhizobiaceae	0.00 ± 0.00	0.00 ± 0.00	1.48 ± 0.18	2.16 ± 0.20
Caulobacteraceae	0.33 ± 0.07	0.02 ± 0.01	1.59 ± 0.23	2.64 ± 0.18
BIrii41	0.27 ± 0.05	0.09 ± 0.09	1.19 ± 0.22	3.86 ± 0.23
Bradyrhizobiaceae	0.47 ± 0.07	0.33 ± 0.03	1.37 ± 0.10	1.51 ± 0.06
Sphingomonadaceae	1.29 ± 0.11	0.67 ± 0.04	4.08 ± 0.25	5.39 ± 0.35

Xanthomonadaceae	1.80 ± 0.18	1.25 ± 0.06	4.05 ± 1.21	5.49 ± 0.71
Enterobacteriaceae	0.00 ± 0.00	0.00 ± 0.00	0.27 ± 0.17	2.07 ± 0.60
Pseudomonadaceae	0.76 ± 0.20	0.24 ± 0.08	4.28 ± 1.25	5.72 ± 1.44
Polyangiaceae	0.00 ± 0.00	0.00 ± 0.00	0.66 ± 0.07	0.60 ± 0.04
Methylophilaceae	0.48 ± 0.13	0.06 ± 0.04	0.30 ± 0.05	1.70 ± 0.20***
Phyllobacteriaceae	0.04 ± 0.03	0.00 ± 0.00	0.79 ± 0.07	1.05 ± 0.12
Actinobacteria	2.58 ± 0.23	1.17 ± 0.16	14.25 ± 0.60	3.44 ± 0.21***
Streptomycetaceae	0.00 ± 0.00	0.00 ± 0.00	9.31 ± 0.77	2.31 ± 0.22***
Pseudonocardiaceae	0.00 ± 0.00	0.00 ± 0.00	1.46 ± 0.36	0.22 ± 0.04***
Bacteroidetes	9.61 ± 0.49	9.01 ± 0.58	10.26 ± 0.88	14.59 ± 0.86***
Cytophagaceae	1.28 ± 0.09	1.42 ± 0.20	1.64 ± 0.26	4.84 ± 0.36***
Flavobacteriaceae	0.65 ± 0.07	0.67 ± 0.12	0.52 ± 0.07	1.99 ± 0.22***
Chitinophagaceae	5.85 ± 0.33	4.60 ± 0.25	4.36 ± 0.35	1.81 ± 0.10***
Sphingobacteriaceae	0.19 ± 0.02	0.25 ± 0.05	3.44 ± 0.44	5.01 ± 0.63
Verrucomicrobia	8.36 ± 0.45	10.04 ± 0.57	1.01 ± 0.19	1.93 ± 0.25
Opitutaceae	0.17 ± 0.04	0.29 ± 0.07	0.25 ± 0.05	0.78 ± 0.11***
Burkholderiaceae	0.01 ± 0.01	0.01 ± 0.01	2.38 ± 0.65	1.16 ± 0.34
Cellvibrionaceae	0.02 ± 0.01	0.04 ± 0.03	0.28 ± 0.03	1.64 ± 0.18***
Chloroflexi	2.32 ± 0.20	1.96 ± 0.13	0.22 ± 0.03	0.55 ± 0.06
Anaerolineaceae	0.90 ± 0.17	0.99 ± 0.21	0.11 ± 0.02	0.44 ± 0.04***
Acidobacteria	29.11 ± 0.77	44.59 ± 1.03	0.92 ± 0.19	0.53 ± 0.08***
Blastocatellaceae	14.08 ± 0.63	28.20 ± 0.91	0.26 ± 0.06	0.13 ± 0.01***
Thaumarchaeota	2.72 ± 0.40	2.44 ± 0.33	0.06 ± 0.01	0.03 ± 0.01***
Firmicutes	0.55 ± 0.07	0.54 ± 0.07	0.14 ± 0.05	0.15 ± 0.03***
Planctomycetes	1.82 ± 0.15	2.08 ± 0.06	0.02 ± 0.01	0.01 ± 0.01***

Table S8. Bacterial collection

A Summary of all the different phyla and families represented by isolates in the bacterial collection. **B** Bacterial isolates of the bacterial collection with the assigned taxonomy using the SILVA database. **C** The 16S rRNA sequence of the different isolates of the bacterial collection. (Additional file 2: Excel sheet)

Table S9. Effect and taxonomy of the screened strains. Increase or decrease in fresh root, shoot, and total weight is indicated as percentage comparing the bacterial treated to the mock treated maize plants; asterisks represent *P* values: * *P* <0.05; ** *P* <0.001; *** *P* <0.001. Two promising PGPR strains were identified and are in **bold.**

Phylum	Family	Genus	Treatment	Repeat	Shoot	Root	Total
Bacteroidetes	*Cytophagaceae*	*Dyadobacter*	RHG21	1	11.2	-0.7	2.7
			RHG21	2	-19.5***	37.3***	9.3
			RHG21	3	98.9***	119.7***	111.8***
	Flavobacteriaceae	*Chryseobacterium*	RHG22	2	30.2***	-7.5	9.2
			RHG22	1	-2.3	-4	-3.3**
			RHG22	2	-6.7	-12.1**	-10
		Flavobacterium	RHG23	2	19.3**	16.8	17.7***
			RHG23	1	-36.2***	-28.7***	-32.3*
	Sphingobacteriaceae	*Mucilaginibacter*	RHG24	3	7.7	-7.4	-1.4
			RHG24	1	-7.3	15	6.4***
		Pedobacter	RHG25	2	21.4*	32.8**	28***
			RHG25	1	-14.9	-25.8***	-21.6
			RHG25	2	9	3.6	5.7*
			RHG26	3	-10.1	-13.7	-12.3***
			RHG26	1	40.2***	71.2***	59.3
			RHG27	2	-20.3*	-8.8	15.9
			RHG27	1	-1.5	-1.7	-1.6
Firmicutes	*Paenibacillaceae*	*Paenibacillus*	RHG28	1	-12.2	38.5***	19
			RHG28	2	14.3	6.1	9.6
Proteobacteria	***Bradyrhizobiaceae***	***Bosea***	**RHG5**	**2**	**32.9***	**23.9**	**28.6***
			**RHG5**	**3**	**5.2**	**25.3****	**15.4***
			**RHG5**	**1**	**1.8***	**18.3**	**12.6***
			RHG6	1	-0.7	6.8	4.6
			RHG6	2	62.7***	59.6***	60.9***
			RHG6	3	-25.1**	-18.5*	-20.5**
	Burkholderiaceae	*Burkholderia*	RHG7	1	-8.9	0.8	-3.9
			RHG7	2	20.4*	-2.7	7.6
			RHG8	2	12	18*	15.3
			RHG8	1	3.4	-20.3**	-8.8
	Caulobacteraceae	*Brevundimonas*	RHG2	1	70.3***	58.5***	63.4***
			RHG2	2	-21.8**	-11.4	-15.8**
			RHG2	3	70.3***	58.5***	63.4***
			RHG3	1	-30.4***	-30.5***	-30.5***
			RHG3	2	-48.1***	-34.2***	-39.6***
			RHG3	3	-44.5***	-39.3***	-41.3***
			RHG4	2	2.5	-1.4	-0.4

		RHG4	1	-3.8	17.9	7.6
	Caulobacter	RHG1	2	32.9***	23.9	28.6***
		RHG1	3	5.2*	25.3***	15.4
		RHG1	1	64.3***	16.6*	32.7***
Comamonadaceae	*Polaromonas*	RHG10	3	-14.6	5.3	-3
		RHG10	1	16.6	12.1	13.8
	Variovorax	RHG9	1	5.5	-1.8	0.6
		RHG9	2	30**	-27***	-1.7
Enterobacteriaceae	*Pantoea*	RHG11	2	3.9	4.9	4.4**
Enterobacteriaceae	*Pantoea*	RHG11	1	-11	-16**	-14.1**
Oxalobacteraceae	***Pseudoduganella***	**RGH12**	**1**	**64.3***	**16.6*****	**32.7*****
		**RGH12**	**2**	**26.7***	**66.2*****	**45.8*****
		**RGH12**	**3**	**-6.5**	**51.8*****	**23*****
Pseudomonadaceae	*Pseudomonas*	RHG13	2	-53.7***	-58.6***	-56.9***
		RHG13	1	19.3	6.3	10.8***
		RHG14	2	38.9**	41.4**	40.5
		RHG14	1	-9.4	-15.7*	-12.6
		RHG15	2	-12.3*	-8.9	-10**
		RHG15	1	-34.4*	13.8***	-11.2*
		RHG16	1	22.7**	0.3	7.6***
		RHG16	2	-27.6***	-18.9**	-21.6***
		RHG16	3	2.9	-13.2	-6.2
Rhizobiaceae	*Rhizobium*	RHG17	1	-2.1	18.8*	12.8
		RHG17	2	43.7**	29.4*	34.8**
Xanthomonadaceae	*Rhodanobacter*	RHG18	1	21.4**	36***	30.3***
		RHG18	2	3	-0.4	1
	Stenotrophomonas	RHG19	2	30.8***	42.8***	38.2
		RHG19	1	-19.4**	-19.8**	-19.6***
		RHG20	2	30.5***	10.8	19.5**
		RHG20	1	1.8	18.3**	12.6**

Table S10: Relative abundance of ID_10 in experiments IV and V. The relative abundance of ID_10 is shown in % (±SE). Asterisks represent *P* values: * *P* <0.05, ** *P* <0.001, and *** *P* <0.001, to indicate a significant enrichment between the bulk soil and the endosphere under chilling or control temperature conditions.

**Sample ID_10**	**Bulk soil**		**Root endosphere**	
	**Control**	**Chilling**	**Control**	**Chilling**
Experiment IV	0 ± 0	0.018 ± 0.018	3.79 ± 0.80 (***)	1.84 ± 0.54 (***)
Experiment V	0.080 ± 0.040	0.023 ± 0.023	3.79 ± 0.80 (***)	1.18 ± 0.11
